# Enhancing the reverse transcriptase function in Taq polymerase via AI-driven multiparametric rational design

**DOI:** 10.3389/fbioe.2024.1495267

**Published:** 2024-12-10

**Authors:** Yulia E. Tomilova, Nikolay E. Russkikh, Igor M. Yi, Elizaveta V. Shaburova, Viktor N. Tomilov, Galina B. Pyrinova, Svetlana O. Brezhneva, Olga S. Tikhonyuk, Nadezhda S. Gololobova, Dmitriy V. Popichenko, Maxim O. Arkhipov, Leonid O. Bryzgalov, Evgeniy V. Brenner, Anastasia A. Artyukh, Dmitry N. Shtokalo, Denis V. Antonets, Mikhail K. Ivanov

**Affiliations:** ^1^ AO Vector-Best, Novosibirsk, Russia; ^2^ AcademGene LLC, Novosibirsk, Russia; ^3^ MSU Institute for Artificial Intelligence, Lomonosov Moscow State University, Moscow, Russia; ^4^ SibEnzyme Ltd., Novosibirsk, Russia; ^5^ Institute of Informatics Systems SB RAS, Novosibirsk, Russia; ^6^ Institute of Molecular and Cellular Biology SB RAS, Novosibirsk, Russia

**Keywords:** bioengineering, function enhancement, reverse transcription, machine learning, protein language model, rational design, Taq polymerase

## Abstract

**Introduction:**

Modification of natural enzymes to introduce new properties and enhance existing ones is a central challenge in bioengineering. This study is focused on the development of Taq polymerase mutants that show enhanced reverse transcriptase (RTase) activity while retaining other desirable properties such as fidelity, 5′- 3′ exonuclease activity, effective deoxyuracyl incorporation, and tolerance to locked nucleic acid (LNA)-containing substrates. Our objective was to use AI-driven rational design combined with multiparametric wet-lab analysis to identify and validate Taq polymerase mutants with an optimal combination of these properties.

**Methods:**

The experimental procedure was conducted in several stages: 1) On the basis of a foundational paper, we selected 18 candidate mutations known to affect RTase activity across six sites. These candidates, along with the wild type, were assessed in the wet lab for multiple properties to establish an initial training dataset. 2) Using embeddings of Taq polymerase variants generated by a protein language model, we trained a Ridge regression model to predict multiple enzyme properties. This model guided the selection of 14 new candidates for experimental validation, expanding the dataset for further refinement. 3) To better manage risk by assessing confidence intervals on predictions, we transitioned to Gaussian process regression and trained this model on an expanded dataset comprising 33 data points. 4) With this enhanced model, we conducted an *in silico* screen of over 18 million potential mutations, narrowing the field to 16 top candidates for comprehensive wet-lab evaluation.

**Results and Discussion:**

This iterative, data-driven strategy ultimately led to the identification of 18 enzyme variants that exhibited markedly improved RTase activity while maintaining a favorable balance of other key properties. These enhancements were generally accompanied by lower K^d^, moderately reduced fidelity, and greater tolerance to noncanonical substrates, thereby illustrating a strong interdependence among these traits. Several enzymes validated via this procedure were effective in single-enzyme real-time reverse-transcription PCR setups, implying their utility for the development of new tools for real-time reverse-transcription PCR technologies, such as pathogen RNA detection and gene expression analysis. This study illustrates how AI can be effectively integrated with experimental bioengineering to enhance enzyme functionality systematically. Our approach offers a robust framework for designing enzyme mutants tailored to specific biotechnological applications. The results of our biological activity predictions for mutated Taq polymerases can be accessed at https://huggingface.co/datasets/nerusskikh/taqpol_insilico_dms

## Introduction

The field of bioengineering has rapidly advanced through the modification of natural enzymes to develop new functionalities and enhance existing properties, addressing critical needs in modern science, industry, and healthcare (Śpibida et al., 2017; Coulther, Stern, and Beuning, 2019; Nikoomanzar et al., 2020; Ouaray et al., 2020). Early on, random mutagenesis followed by selection that mimics natural evolution was the most widespread way to obtain new mutated enzymes with modified functions of interest. This approach, while being fruitful, is time-consuming and labor-intensive. Later, rational-design approaches began to emerge, based on information obtained from protein alignments and structures of their complexes with ligands. These approaches, substantially advancing enzyme engineering, are often based on extensive empirical data and can be limited by the complexity of protein interactions. As the quest for efficiency and precision in protein engineering continues, the integration of computational tools has become inevitable.

Deep learning, especially involving the use of language models, has transformed protein science by enabling researchers to efficiently harness vast genomic databases like BFD and UniRef50. These protein language models (PLMs), trained via unsupervised pretraining techniques, predict masked amino acids by interpreting contextual information from visible sequence data. The resulting embeddings—dense, information-rich vectors for each amino acid—capture essential biophysical and structural properties not explicitly mentioned in the data. Through aggregation of these embeddings, entire protein sequences can be parameterized, laying a comprehensive foundation for predicting mutations’ effects on a protein’s structure. Such capabilities allow for practical application of these models in various bioengineering tasks, including prediction of secondary structures, of residue contacts, and of a mutational impact on enzyme functionality (Elnaggar et al., 2022; Rives et al., 2021; Lin et al., 2023; Notin et al., 2022; Rao et al., 2021; Jumper et al., 2021), thus advancing the field beyond previously available methods.

The first remarkable application of a PLM to rational enzyme engineering was achieved with UniRep (Alley et al., 2019), which was trained on over 20 million protein sequences from UniRef50, thereby allowing the model to learn general protein features in an unsupervised manner. The representations learned by UniRep effectively support linear regression models in guiding directed evolution, thus proving adequate for capturing necessary information about mutant proteins (Alley et al., 2019). It has also been found that UniRep simulates a fitness landscape accurately enough for engineering applications using only 24 functionally characterized proteins bearing amino acid substitutions (Biswas et al., 2021). UniRep utilizes a deep recurrent neural network based on the biLSTM architecture, which is relatively small by modern standards. In contrast, current large language models, especially those in protein science, predominantly involve the transformer architecture, which has been demonstrated to be superior in handling complex sequence data. Among modern families of PLMs, one can mention ESM (Verkuil et al., 2022; Hie et al., 2022), RITA (Hesslow et al., 2022), ProtT5 (Elnaggar et al., 2022), ProstT5 (Heinzinger et al., 2023), ProGen2 (Madani et al., 2023), ProtGPT2 (Ferruz et al., 2022), and ECNet (Luo et al., 2021), representing the current state of the art in PLMs and yielding promising results in various applications including protein design. Alongside the primary use of PLMs for predicting protein behaviors, multiple sequence alignment (MSA)-based models, also known as MSA transformers (Rao et al., 2021; Jumper et al., 2021), introduce a unique approach to integrating evolutionary information during protein analysis. These models require constructing MSAs as input, thereby effectively taking two-dimensional (2D) input instead of traditional 1D data. This 2D input necessitates a specialized architecture, significantly increasing computational and memory demands.

In the majority of enzyme bioengineering studies, candidate enzymes have been selected based on a limited set of parameters from a huge number of mutants obtained by randomized mutagenesis. The latter typically generates a large pool of candidates, which has to be investigated in detail to choose the most promising mutants. Given that the evaluation of an original pool of candidates by wet-lab experiments is time-consuming and labor-intensive, initial sorting of candidates is traditionally done in a limited series of experiments. If the candidate must simultaneously exhibit several useful properties, the limitation on the scope of experiments conflicts with full-fledged evaluation. As a consequence, possible negative effects of the introduced mutations on some important properties of the modified enzyme can be overlooked, and, on the other hand, the main improved characteristic can be evaluated under suboptimal conditions. For this reason, some promising candidates can be rejected by the initial selection, and conversely, candidates with undesired properties can pass through the selection screen because the same amino acid substitution at a functionally significant position can simultaneously affect several functions, sometimes in opposite directions.

In this regard, in our study, we suggest an alternative strategy for a multifunctional enzyme design: *in silico* preselection of candidate mutants by means of rational predictions about an extended range of biotechnologically significant properties, to take them into account right from the start. We believe that the iterative procedure involving several rounds of computational prediction and subsequent experimental studies must be a promising alternative to the traditional approaches, especially when tradeoffs must be made between several simultaneously required biological functions and physicochemical characteristics. To prove this concept, we attempted to construct and experimentally validate a predictive model aimed at selecting mutants of *Thermus aquaticus* DNA polymerase (hereinafter referred to as Taq pol)—having a set of required properties—by combining structure-based rational design and experimental results of multiparametric wet-lab assays.

Taq pol, a thermostable DNA-dependent DNA polymerase, has found widespread applications (primarily in amplification techniques) due to a combination of numerous useful properties, such as ease of accumulation in bacterial expression systems, extreme thermostability, high processivity, and strong 5′–3′ exonuclease activity. Therefore, Taq pol is a good example of an enzyme that requires a combination of several different activities to be successfully used as a tool in molecular biology and biotechnology. Wild-type Taq pol is characterized by relatively low fidelity (because it lacks an active 3′–5′ exonuclease proofreading domain), negligible strand displacement activity, marked decrease in activity during the insertion of non-canonical substrates, and has only very low intrinsic RNA-dependent DNA polymerase activity. This limits the use of wild-type Taq pol as a core enzyme in some molecular biological applications. At the same time, Taq pol is known for its structural plasticity: it tolerates multiple amino acid substitutions, even in evolutionarily conserved regions (Loh and Loeb, 2005). This property makes Taq pol a convenient model for function enhancement and modification studies. Numerous modified versions of Taq pol have been obtained that are characterized, for example, by increased resistance to PCR inhibitors (Kermekchiev et al., 2009), improved elongation (Yamagami et al., 2014) and strand displacement abilities (Ignatov et al., 2014; Barnes, Zhang, and Kermekchiev, 2021), 3′–5′ exonuclease activity (Park et al., 1997), a reduced capacity to discriminate against dideoxynucleotides (Laos, Thomson, and Benner, 2014) or to elongate mismatched PCR primers (Drum et al., 2014; Lim et al., 2022), wider substrate specificity (Ghadessy et al., 2004; Schultz et al., 2015), or cold sensitivity (Kermekchiev, Tzekov, and Barnes, 2003).

One of Taq pol engineering directions is the creation of enzymes with dual DNA- and RNA-dependent DNA-polymerase (reverse transcriptase, RTase) activities for biotechnology, molecular genetic studies, and applied diagnostic tools. Traditional mesophilic RTases such as M-MuLV or AMV have difficulty synthesizing cDNA through stable RNA secondary structures and GC-rich motifs. High-temperature reverse transcription (RT) may enhance cDNA synthesis efficiency and reduce primer dimerization and side product formation. RTases derived from Taq pol are expected to retain these properties and thus to have an advantage over mesophilic RTases. The single-enzyme approach could streamline the development of real-time RT-qPCR tools, also benefiting from the enzyme’s thermostability and inhibition tolerance in terms of storage, shipping, and automation. These and other potential benefits stimulate the ongoing efforts to create new high-temperature RTases engineered from Taq pol and other thermostable DNA polymerases. Successful examples of enhancement of the RTase activity in Taq pol via introduction of one or more point mutations have long been known (Sauter and Marx, 2006; Ong et al., 2006; Vichier-Guerre et al., 2006; Marx et al., 2010; Blatter et al., 2013; Raghunathan and Marx, 2019; Barnes, Zhang, and Kermekchiev, 2021; Huber, Betz, and Marx, 2023). Of note, in different studies, modifications at completely different sites have had similar effects on this property. For instance, in ref. (Vichier-Guerre et al., 2006), a directed-evolution experiment with the Stoffel fragment of Taq pol gave 27 mutant enzymes showing appreciably (up to two orders of magnitude) enhanced RTase activity, which was achieved for different mutants through amino acid substitutions at more than 50 positions.

In our study, we set the following selection goal: to create an enzyme that a) possesses an enhanced RTase activity in a PCR-compatible buffer, and b) retains some beneficial properties of the wild-type enzyme (sufficient fidelity, 5′–3′ exonuclease activity, thermal stability, the ability to effectively incorporate deoxyuracyl, and the capacity to process locked nucleic acid (LNA)-containing substrates; hereafter: LNA substrates) that are unaffected or even improved. A list of parameters was compiled based on the suitability of mutants for molecular diagnostic applications. Additionally, we tested all the produced mutant enzymes for the “hot start” capacity through blocking by a monoclonal antibody or a DNA aptamer. By means of several rounds of experiments, we created a set of multivariate regression models built on top of embeddings obtained via the ProtT5 language model family (Elnaggar et al., 2022). This PLM-based regression model efficiently integrated the evolutionary insights and allowed us to identify several Taq pol mutants with noticeably enhanced RTase activity combined with the preservation (or even enhancement) of a number of required preselected characteristics.

## Materials and methods

The selection of the first batch of Taq pol mutants was guided by literature data to ensure a diverse range of physicochemical changes, such as variations in charge, volume, aromaticity, and polarity, which could influence enzyme function in different ways (see details in the *Results* section).

### Generation of Taq pol mutants

The nucleotide sequence of the Taq pol–encoding gene was codon-optimized for translation in the *Escherichia coli* expression system. All rare codons were eliminated, and codon frequency after the optimization was equal to or greater than 8/1,000. The coding part of the synthetic gene sequence started from the fourth codon of the original Taq pol sequence: therefore, the protein we used differed from native Taq pol by the absence of the first 3 amino acid (aa) residues. Nonetheless, to facilitate the comprehension and interpretation of the results in the context of publications by other research groups, the numbering of amino acid positions corresponding to native Taq pol is utilized throughout the text. Oligonucleotides for PCR-based gene synthesis were designed in online software DNAWorks (v3.2.4) (https://hpcwebapps.cit.nih.gov/dnaworks/). The gene sequence was divided into five fragments ∼500 bp long and flanked with restriction endonuclease sites, which helped to clone the gene into the pJET1.2/blunt vector (Thermo Fisher Scientific, United States). The mutations were introduced into the corresponding fragments by site-directed mutagenesis. The full-length gene for each mutant was assembled by sequential ligation of fragments into the pET28a-Novagen expression vector (Sigma-Aldrich, United States). All sequences of synthetic constructs were verified by Sanger sequencing on an Applied Biosystems 3,500 instrument (Thermo Fisher Scientific) with the BigDye Terminator v3.1 Cycle Sequencing Kit. All Taq pol mutants were expressed in *E. coli* BL21 (DE3)pLysS. Competent cells were transformed with the expression plasmid and grown at 37°C in 400 mL of the Luria–Bertani medium containing 30 μg/mL kanamycin and 34 μg/mL chloramphenicol. When cell density reached OD600 of 0.8–0.9, protein expression was induced with 1 mM isopropyl β-D-1-thiogalactopyranoside. After 3 h of expression, the cells were centrifuged at 6,400 g for 10 min at 4°C and stored at −70°C until analysis. Cell pellets were resuspended in a buffer consisting of 20 mM NaH_2_PO_4_ (pH 8.0), 50 mM NaCl, 0.1 mM EDTA, 1 mM PMSF, and 1% of Triton X-100. After heat denaturation at 75°C for 40 min in a water bath, the lysates were centrifuged at 39,000 × g and 4°C for 20 min. The supernatant was treated with 0.05% polyethyleneimine to remove chromosomal DNA and centrifuged at 39,000 × g and 4°C for 20 min. The supernatant was passed through a 0.45 μm syringe filter and purified on a Ni-NTA column using a buffer composed of 20 mM NaH_2_PO_4_ (pH 8.0), 0.5 M NaCl, 0.1 mM EDTA, 15 mM imidazole, and 0.1% of Triton X-100. The proteins were eluted with 20 mM NaH_2_PO_4_ pH 8.0, 0.5 M NaCl, 0.1 mM EDTA, 250 mM imidazole, and 0.1% of Triton X-100, and then dialyzed against another buffer (20 mM Tris HCl pH 8.0, 75 mM NaCl, and 0.1 mM EDTA) overnight with stirring in a cold room. The concentration of purified proteins was determined spectrophotometrically.

### Agarose gels

Horizontal electrophoresis chamber Wide Mini Sub Cell GT and Gel Documentation System Gel Doc XR+ (Bio-Rad, United States) were used to examine 2% agarose gels during electrophoresis and to take photographs.

### Oligonucleotide primers and probes

The oligonucleotides were designed using the PrimerQuest online service (https://eu.idtdna.com/calc/analyzer). All oligonucleotides were synthesized at Vector-Best (Russia). Structures of primers and fluorescently labeled probes are presented in Supplementary Table S1.

### Determination of equilibrium dissociation constants

Kinetic curves of the change in fluorescence anisotropy in kinetic assays of the formation of enzyme–substrate complexes (determination of equilibrium dissociation constants: K_d_) as well as kinetic curves of the change in SYTO 13 fluorescence in experiments on the rate of polymerase-driven synthesis [determination of catalytic constants of the polymerization reaction rate in the presence of dT and dU: k_Cat_(dT) and k_Cat_(dU)] were recorded on an SX20 stopped-flow spectrometer (Applied Photophysics, United Kingdom) with the Pro-Data SX20 software (Applied Photophysics). Each kinetic curve was obtained by averaging 15 to 30 experimental curves. The fluorescence anisotropy measured in the experiment at each time point depends on the current concentration of the complex of polymerase with a labeled substrate called H-Kd (see Supplementary Table S1): the higher the concentration of the specified complex, the higher the anisotropy value is. Quantitative processing of the experimental data was carried out using the “minimize” function of the scipy. optimize Python library via optimization of the parameters determining the formation of the “polymerase-labeled oligonucleotide” complex (in the case of K_d_ calculation) and through optimization of the parameters determining the elongation rate of the 20_60 hairpin template [in the case of k_Cat_(dT) and k_Cat_(dU) calculation]. Reaction conditions were as follows: cell volume, 20 μL; temperature, 55°C; buffer: 5 mM Tricine-KOH pH 8.0, 100 mM KCl, 3.4 mM MgCl2, 0.1 mM each dNTP, 0.01% of Tween 20, 1/5000 SYTO 13, and 20 nM hairpin template. Fluorescence was detected at an excitation wavelength of 535 nm and a cutoff filter >570 nm (in the case of K_d_ determination) or using an excitation wavelength of 485 nm and a cutoff filter >530 nm [in the case of k_Cat_(dT) and k_Cat_(dU) determination].

### Assessment of the hot-start capacity by means of a monoclonal antibody and DNA aptamer

This experiment with a monoclonal antibody to Taq pol (Clontech, United States) and a DNA aptamer (Vector-Best) was performed as described elsewhere (Bragin et al., 2008). Briefly, polymerase kinetics and their change related to the blocking by the antibody or aptamer were registered by means of an increase in SYTO 13 fluorescence after elongation of the OnOff hairpin template (see Supplementary Table S1) during catalysis by different concentrations of tested enzymes at different temperatures. Hereinafter (if not specified otherwise), in all kinetic assays, a 1/2,500 dilution of SYTO 13 from a commercial stock solution (Thermo Fisher Scientific) was used. The reaction was carried out in 1× RT buffer (50 mM tricine-KOH pH 8, 50 mM KCl, 0.0001% of Tween 20, 100 μg/mL BSA, and 3 mM MgCl_2_) with 0.4 mM each dNTP and 100 nM OnOff hairpin template as 500 cycles at 45°C. Blocking was considered effective when it resulted in at least a 10-fold decrease in the reaction rate.

### The effective reaction rate constant evaluation

The effective reaction rate constant was evaluated under the same conditions as in the hot-start experiment, via 500 cycles at 60°C. For each enzyme, four concentrations were analyzed in serial 2-fold dilutions. Prior to this, the lowest concentration of each enzyme was experimentally found that gave a kinetic curve of the classic shape. Each enzyme concentration was analyzed in triplicate, and the experiment with each enzyme was conducted twice: thus, for each tested concentration of each enzyme, six kinetic curves were built. The inverse kinetics problem was examined for each curve dataset via calculation of the effective reaction rate constant. The inverse problem was solved by a numerical approach involving Gauss and RKM methods for a system of differential equations and “the Peak descent” method for step-by-step curve approximation (Tomilov, Zagoruiko, and Kuznetsov, 1999). The effective rate constants obtained in this way were averaged for each enzyme. Finally, the ratio of the activity of each mutant enzyme to the control was computed:

$$R\left(ratio\right)=\frac{k_{mut}^{eff}}{k_{cont}^{eff}}$$

### The effect of dTTP substitution with dUTP on the DNA synthesis rate

The influence of dTTP substitution with dUTP on the synthesis rate of the DNA-dependent DNA polymerase was evaluated under the same conditions. The second and third concentrations of each enzyme from the serial dilutions described above were tested. Each enzyme concentration was analyzed in triplicate. The experiment with each enzyme was conducted in two ways: either with 0.4 mM each dNTP including dTTP or with dUTP instead dTTP at the same concentration. Data were recorded as the ratio of effective reaction rate constants between reactions in dTTP-containing buffer and dUTP-containing buffer (hereinafter: the dT/dU rate).

### Tolerance to LNA substrates

Evaluation of the ability to process LNA substrates was performed under the same conditions. The second concentration for each enzyme from the serial dilutions described above was utilized. Each enzyme was tested in triplicate with each of four DNA hairpin substrates: LNA-0, LNA-1, LNA-2, and LNA-3 (all of which had identical structure, but LNA-1–3 carried LNA nucleotides at different positions imitating the LNA modifications of PCR primers and DNA templates, see Supplementary Table S1; Supplementary Figure S1). The kinetic curves were averaged for each enzyme/substrate pair. For each enzyme, the ratio of the extension rate of the LNA-modified hairpin to the extension rate of the substrate without modification was evaluated. The closer this ratio was to 1, the more tolerant the enzyme was to this LNA modification variant.

### Reverse transcription activity assessment

RTase activity was assessed for all Taq pol variants in two ways.

1) Through direct evaluation of RT kinetics by means of the increase in SYTO 13 fluorescence during extension of a double-stranded substrate comprising 7.5 nM oligo (dT) primer and 600 pg/μL poly(r)A. The 50 μL reaction was carried out in 1× RT buffer with 14.5% of trehalose, and 240 μM dTTPs on a CFX96 thermocycler with an optical module (Bio-Rad) by the following protocol: 500 cycles at 45°C. Enzyme concentrations were selected so that the kinetic curve could be clearly recorded and had the classic shape. Each experiment was conducted in duplicate, and curves plotted from the mean values for each experimental data point were employed for interpretation. Ratios of the activity of each mutant enzyme to controls (which were the WT and subunit p66 of HIV RTase, hereinafter referred to as p66 (Vector-Best) were computed as described above.
2) By assessing the relative synthesis efficiency of four specific cDNAs of different lengths compared to natural reverse transcriptase. The RT (30 min at 60°C) was allowed to proceed in a 50 μL reaction mixture composed of 1× PCR buffer (Vector-Best) supplemented with 10% of trehalose, 100 μg/mL BSA, 5 mM MgCl_2_, 0.4 mM each dNTP, 0.6 M betaine, and 500 nM corresponding sequence-specific reverse primer (see Supplementary Table S1). Synthetic RNA transcripts containing specific sequences of the human parainfluenza virus 2 (HPIV2) phosphoprotein gene (90 nt), human metapneumovirus (hMpV) nucleoprotein gene (116 nt), rhinovirus (RhV) 5′-UTR (201 nt), and human immunodeficiency virus (HIV-1) 5′ LTR (526 nt) were added at the same concentration (∼10^6^ copies/reaction) to spike the reaction mixture. p66, serving as the reference enzyme, was assayed under the same conditions, except for the temperature and enzyme concentration, which were selected in preliminary experiments (15 nM and 50°C). 5 μL of each RT reaction were mixed with respective sets of primers and fluorescently labeled probes (See Supplementary Table S1) in nucleic acid elution buffer (Vector-Best) added to the final volume of 50 μL, and then amplification was performed by real-time PCR with the help of ready-to-use freeze-dried master mixes containing PCR buffer and the wild-type Taq pol (Vector-Best). The thermal cycling protocol was as follows: 2 min at 95°C and then 50 cycles of 10 s at 94°C and 20 s at 60°C. Amplification data were recorded on the CFX96 instrument. Each experiment was conducted twice, and the obtained Cq values were averaged. RTase activity in each “Taq pol mutant/cDNA” pair was assessed as the difference between the average Cq value shown by the tested enzyme and the value shown by the p66.

The second method turned out to be more informative and reproducible, and it was the results obtained by it that were used to train models.

### Assessing the usability of selected Taq pol mutants in a single-tube RT-PCR with TaqMan detection

Enzymes with the best combination of desirable characteristics (sufficient DNA-dependent DNA polymerization rate and PCR efficiency, enhanced RTase activity, the ability to utilize LNA substrates and dUTP, and the capacity to effectively cleave TaqMan probes) were tested for suitability for single-tube RT-PCR. The 50 μL reaction was carried out on the CFX96 thermocycler in 1× PCR buffer with primers to the target sequences at a concentration of 500 nM each and fluorescently labeled probes at 250 nM. Each mutant enzyme was added at 120 nM, and synthetic RNA transcripts carrying the 90-nt HPIV2 sequence and the 116- nt hMpV sequence were used to spike the reaction mixture at 10^6^ and 10^4^ copies/reaction. Either a mixture of 26 nM p66 and 120 nM WT or 120 nM WT alone served as a control. The following thermal cycling conditions were implemented: 15 min at 60°C (found for all mutant Taq pol mutants in preliminary optimization experiments) or 15 min at 50°C (for the p66), then 1 min at 94°C and 50 cycles of 10 s at 94°C and 20 s at 60°C with fluorescence recording. The data were interpreted with respect to two parameters: Cq reflected integral efficiency of RT and cDNA amplification, and the amplitude and shape of the fluorescence curve signified the 5′ exonuclease activity resulting in the cleavage of the labeled probe.

### DNA-dependent DNA polymerase synthesis fidelity assessment

The fidelity of DNA-dependent DNA polymerase activity of each polymerase variant was evaluated as the proportion of misincorporated nucleotides during synthesis of a specific 99 bp fragment of the hepatitis B virus (HBV) S gene with this enzyme in PCR. The fidelity values were calculated from the data obtained by next-generation sequencing of the PCR products. Purified HBV DNA (7.5 pg/reaction) served as a template for PCR. HBV genome fragments were synthesized on Bio-Rad CFX96 in a volume of 30 μL of 1× PCR buffer with primers HBV-F and HBV-R at 500 nM each and the fluorescently labeled HBV-P probe at 250 nM according to the following protocol: 2 min at 95°C, then the cycles: 10 s at 94°C, and 20 s at 60°C. Taq pol mutants were added at 120 nM. The reaction was stopped at the time of reaching a plateau (for different enzymes, it required different number of amplification cycles). PCR was monitored in real-time using SYTO 13 fluorescence (to track the reaching of a plateau) and ROX fluorescence (to assess PCR efficiency and the ability to cleave 5′-labeled probes). It is noteworthy that the PCR efficiency determined in this analysis was applied as an independent criterion to model training and selection of candidates.

PCR products were purified by SPRI on Ampure XP paramagnetic microbeads (Beckman Coulter, United States) prior to library construction and quantified on a Qubit 2.0 instrument with the help of the dsDNA High Sensitivity Quantitation kit (Thermo Fisher Scientific). A purified PCR product (50 ng) was used for construction of NGS libraries by the NEBNext^®^ Ultra™ II DNA Library Prep Kit for Illumina (New England Biolabs, United States). KAPA UDI Primer Mixes (KAPA biosystems, cat. No. 09134336001) were employed for library indexing to minimize the risk of library cross-contamination. Illumina adapters were ligated to the resultant fragments and barcoded by PCR with primers from the NEBnext multiple oligos for the Illumina kit (New England Biolabs, cat. #E7600) and Phusion DNA polymerase (Thermo Fisher Scientific). Sequencing was performed on the Illumina MiSeq 2,500 instrument (Illumina, United States). The coverage per sample was 88–698 thousand 150-nt paired-end reads. To map reads to the HBV genome and identify nucleotide substitutions in them, the UGENE v42.0-dev software package (Unipro, Russia) and the BWA-MEM and SAMtools tools built into it were used. To reduce the probability of mistaking sequencing errors for the errors of a tested polymerase at the stage of selecting variants, all reads of individual nucleotides (either matching the reference or different from it) with a Q-value >30 (the probability of sequencing error is >0.01%) were discarded. Further calculations were performed in Microsoft Excel 2019 (Microsoft, United States). All detected nucleotide substitutions that represented a deviation from the reference and were not part of the primers were designated as polymerase errors. First, for each nucleic-acid sample, the frequency of nucleotide substitutions in the final pool of amplicons was evaluated as:

$$f=N_{\mathrm{alt}}/N_{\mathrm{alt}+\mathrm{ref}}$$

where ***f*** is the average frequency of nucleotide substitutions for all positions, ***N***_***alt***_ is the number of nucleotide reads that differed from the reference (total for all positions of the target), and ***N***_***alt+ref***_ is the total number of nucleotides read (total for all positions of the amplicon). The polymerase fidelity was then calculated from the error rate via the formula:

p=E×f/n

where ***p*** is the error rate, ***f*** denotes the error rate in the final amplicon population, ***E*** is PCR efficiency, and ***n*** represents the number of amplification cycles. The efficiency of each individual reaction was assessed by means of the fluorescence curve shape in the LinRegPCR software (Ruijter et al., 2009). For each enzyme, the experiment was conducted in triplicate, and the resulting fidelity assessment was the averaged data.

### Parametrization of protein sequences

In our work, we employed transformer-based PLMs to parametrize mutated protein sequences into dense vectors. In particular, we utilized the encoder part of the encoder-decoder ProtT5-XL model (Elnaggar et al., 2022), using last-layer embeddings of the encoder for parametrization. The resulting per-token sequence embeddings were next aggregated by average pooling to obtain sequence level representations. These served as input to the predictor on top of the language model embeddings.

We fine-tuned the last six layers of the ProtT5-XL encoder using the masked language modeling objective because the authors of ref. (Biswas et al., 2021) argue that fine-tuning on homologs of a target protein sequence greatly improves the results of protein function prediction models, and our experiments suggested the same, though the effect was less drastic for larger models.

We chose the model implementation released in the HuggingFace library (Wolf et al., 2020) and fine-tuned it with ZeRo stage 2 optimization (Rajbhandari et al., 2020) available in the DeepSpeed library by Microsoft (https://github.com/microsoft/DeepSpeed). The model was fine-tuned for 2 weeks on a server containing four NVIDIA V100 GPUs at a batch size of 1,024 and a learning rate of 1e-5, with a linear decay schedule, and 200 warmup steps.

The data employed to fine-tune the model were retrieved from the UniRef100 database. Homologous sequences were extracted using jackhmmer (Eddy, 2011) with default settings and the Klenow fragment of Taq pol (UniProt ID: *P19821*) as a reference. At the time of data acquisition (January 2022), the UniRef100 database contained 280,483,851 proteins. The extracted set of homologous proteins consisted of 91,808 sequences with lengths up to 949 aa (mean length of 703.3 and a median of 875 aa).

### Regression models

In this project, we employed regression models on top of embeddings to predict functional effects of amino acid substitutions in Taq pol. For the first batch of data, we utilized the Ridge regression to capture a relation between the embeddings and the target properties. The ridge regression model was chosen for its simplicity and effectiveness in handling multicollinearity among features. We fine-tuned the α parameter by leave-one-out cross-validation, with mean absolute percentage error (MAPE) as the evaluation metric.

For the second batch of data, we switched to Gaussian Processes using the GPytorch library (Gardner et al., 2021). This decision was driven by the limitations observed in linear models, which sometimes produced extreme predictions. Gaussian Processes have the additional advantage of quantifying the uncertainty of predictions, thereby offering a more nuanced understanding of a model’s confidence in its outputs.

Each property was modeled using manually selected kernels and their respective parameters. We primarily utilized Matern or SpectralDelta kernels and adjusted the number of deltas as needed to best capture the underlying data patterns. This manual selection process ensured that the kernels were well-suited to specific characteristics of each property, thereby enhancing a model’s predictive performance.

To prepare the targets for the modeling, all values were scaled by subtraction of the mean and dividing by the standard deviation. Additionally, some targets were logarithmically transformed beforehand to stabilize variance and normalize the distribution. After predictions were made by Gaussian Processes, the density of the predictions was transformed back to the nonlogarithmic space in order to maintain interpretability. All parameters and their configurations can be found in the Supplementary Table S2.

### Selection and validation of predictive models

Lacking direct experimental data for Taq pol, we availed ourselves of deep mutational scanning data from BlaC (Firnberg et al., 2014) and avGFP (Sarkisyan et al., 2016) for benchmarking, which later became a part of the ProteinGym benchmark (Notin et al., 2023). The latter provides extensive mutational data for over 200 proteins, making it beneficial for this research field.

For model selection, we performed repeated random sampling of small training sets, which allowed us to estimate the distribution of metrics by means of the remaining data. This approach reflects our initial setting of small training sets (e.g., 18 mutants) for Taq pol. We evaluated model performance as Spearman’s correlation coefficients and a set of Top-k metrics adapted from (Biswas et al., 2021). Spearman’s correlation—a rank-based metric—was chosen to evaluate the ordinal relation between predicted and actual activity values because our primary goal was to rank mutants by functionality rather than predict exact values. Top-k metrics (k = 4, 8, 16, and 24) were used to describe the hits that exceeded the WT in terms of functionality among the top predictions. These metrics are especially useful in real-world scenarios where the selection of enhanced mutants is based on a model’s top predictions, and the extent of wet-lab experimental validation is limited. The fine-tuned ProtT5-XL yielded the results comparable to those of non-fine-tuned ProtT5-XXL, and we selected the ProtT5-XL model for protein parametrization owing to its smaller size.

### Mutational scanning at the chosen sites

The selection of the mutation set for our study was guided by several critical considerations. Firstly, we limited the number of substitutions per mutant to no more than three. This approach was adopted to avoid scenarios where a substitution can disrupt useful properties of the enzyme and this phenomenon may go undetected by our regression models. These models, designed to simulate a fitness landscape, may not accurately predict such problems owing to being trained on relatively sparse data. To refine our mutation site selection procedure, we relied heavily on insights from pertinent literature. Our literature review was instrumental in narrowing down the search space; exhaustively searching for all possible triple substitutions across all Taq pol mutants would have resulted in over 1,011 data points. Consequently, our mutational scanning encompassed all single mutants at every position in Taq pol along with double and triple mutants specifically at aa 507, 515, 536, 540, 570, 573, 578, 586, 614, 626, 639, 670, 667, 707, 708, 728, 732, 742, 743, 747, and 783. This targeted approach enabled us to efficiently explore mutations having the potential to alter the enzyme’s properties in line with our study’s goals.

**FIGURE 1:**
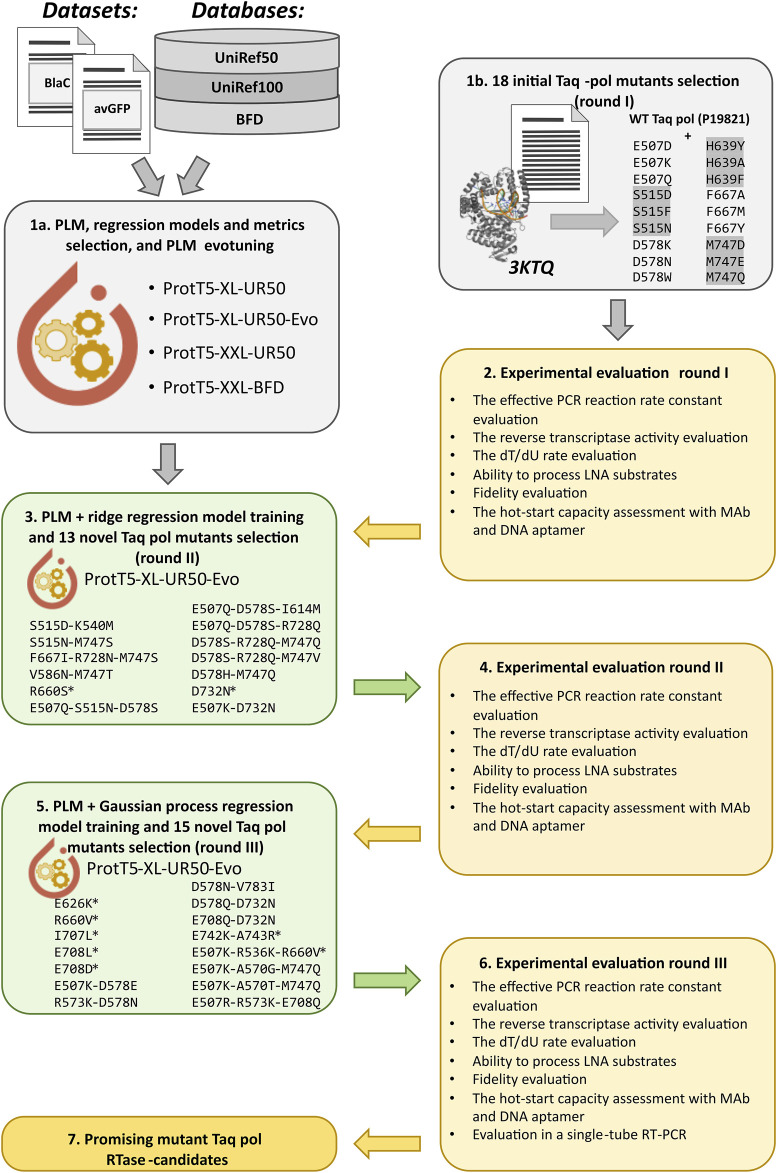
The outline of the Taq pol variants design and evaluation pipeline. First, the *in silico* analyses of PLMs and different regression models for protein function enhancement were performed with published mutational data, and the chosen PLM was fine-tuned with Taq pol homologs (evotuned) (1a); and the initial aa substitutions were selected for wet-lab experimental evaluation based on structural analysis and literature sources (1b). Then, the first round of experimental evaluation was performed (2), and the evotuned ProtT5-XL-UR50-Evo model was used to obtain the embeddings of Taq pol and a vast set of its mutants with 1–3 aa substitutions, after which the first regression model was built, and a new set of Taq pol mutants was selected (the mutated variants marked with asterisk were selected from literature sources) for validation (3). After the second round of experiments (4), the second model was obtained based on ProtT5-XL-UR50-Evo and Gaussian Process regression, and another set of Taq pol mutants was chosen (5) for the third round of experimental assessment (6).

An outline of the Taq pol variants design and evaluation pipeline is given in Figure 1.

## Results

### Starting the selection of substitution mutants

To train the first version of the model aimed at predicting effects of amino acid changes on the selected Taq pol parameters, we began with a multiparametric wet-lab testing of a limited set of mutants carrying various single amino acid substitutions that were located at key positions in different structural domains and were expected to affect various protein properties in different directions. Initially, we applied the following restrictions: for resource-saving reasons, to characterize only 18 mutants first, together with the wild-type Taq pol (hereinafter referred to as the WT) serving as a reference; to check several key positions localized to all structural domains (thumb, palm, and finger); and to include a few mutants that have low expected activity in PCR. Thus, using a limited number of options, we intended to obtain various combinations of substitutions in terms of changes in such physicochemical properties as charge loss/replacement, physical volume, aromaticity, and polarity.

**FIGURE 2:**
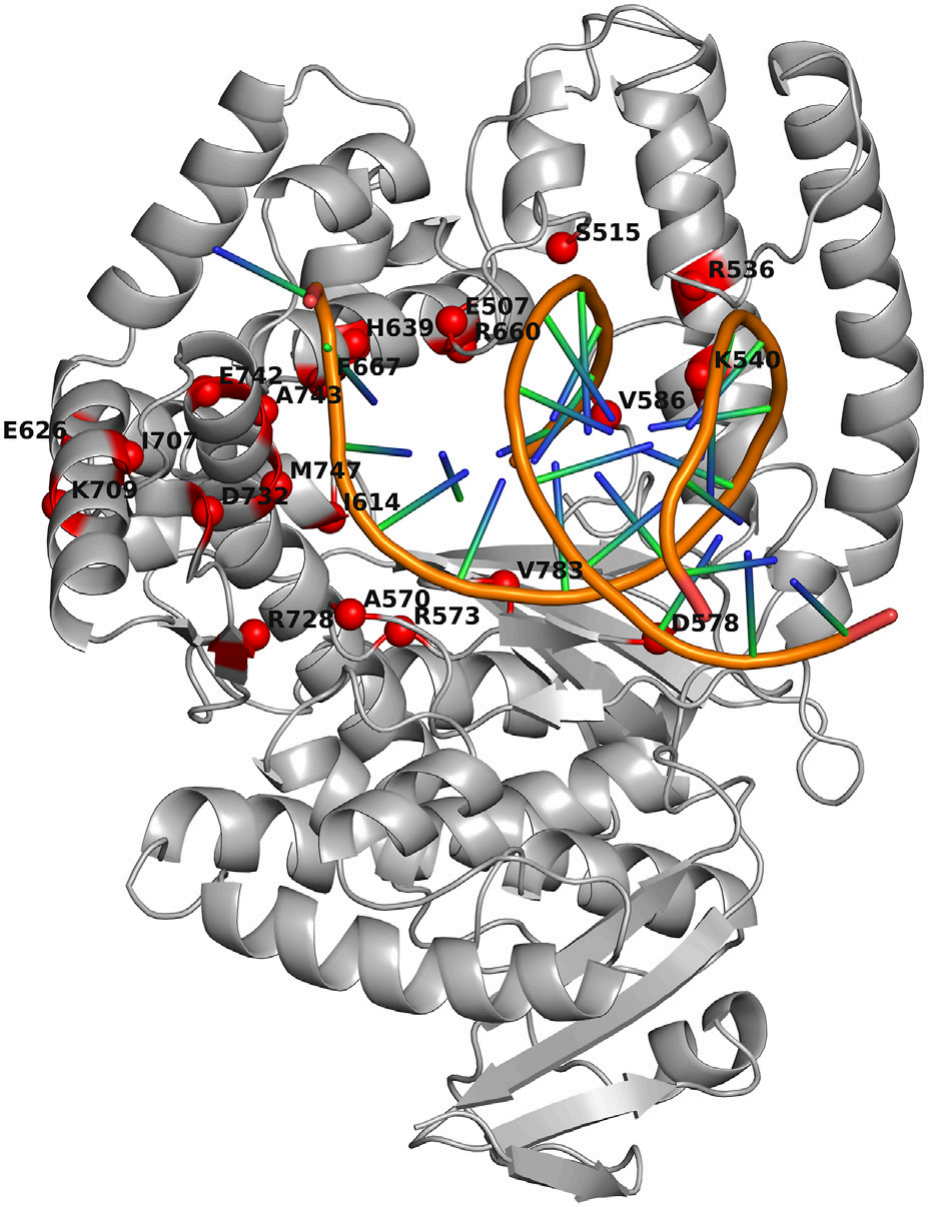
Locations of assayed amino acid positions in 3D structure of the large fragment of *Thermus aquaticus* DNA polymerase I complexed with a DNA molecule (Protein Data Bank ID: *3KTQ*). The spatial structure of Taq pol is presented as a gray ribbon diagram. Red spheres denote Cα atoms of amino acid residues (aa) that were mutated alone or in several combinations. The labels indicate residues and their positions in WT Taq pol (SwissProt: *P19821*). The image was produced in PyMOL v.2.5.0 (Schrödinger and DeLano, 2021).

The results from (Raghunathan and Marx, 2019) were employed as a starting point for choosing the substitutions to be evaluated experimentally. There, 12 key positions were investigated in detail: N483, E507, S515, and K540 (thumb); A570, D578, V586, and V783 (palm); and I614, H639, F667, and M747 (finger). We selected six out of these 12 positions: M747 and D578 (contacting the template strand), E507 and S515 (coming into contact with the primer strand), and H639 and F667 (binding to an incoming nucleoside-5′-O-triphosphate). Other positions were excluded due to their high tolerance to amino acid substitutions. Thus, the following 18 mutations were finally chosen for the first round of wet-lab assays: E507D, E507Q, E507K, S515D, S515F, S515N, D578K, D578N, D578W, H639A, H639F, H639Y, F667A, F667Y, F667M, M747E, M747D, and M747Q. Six conservative substitutions (E507D, E507Q, S515D, D578N, H639Y, and F667Y) and four substitutions expected to result in PCR activity loss (S515F, H639A, F667A, and M747D) according to ref. (Raghunathan and Marx, 2019) were intentionally included in this list. Locations of the investigated amino acid positions in 3D structure of the Taq pol large fragment complexed with a DNA molecule are depicted in Figure 2 (together with other positions further assayed in our study).

### Multiparametric testing of the first 18 mutant polymerases

The results of testing of the first 18 mutant enzymes in comparison with the WT enzyme are summarized in Table 1. For the WT, the results were comparable to those in the literature. Despite the expected loss of PCR activity in four mutants (S515F, H639A, F667A, and M747D) according to (Raghunathan and Marx, 2019), in our experiments all the enzymes manifested the DNA-dependent DNA polymerase activity and the ability to perform real-time PCR with cleavage of fluorescently labeled probes. Nevertheless, in all cases, the rate of this synthesis was found to be reduced as compared to the WT. According to the rate of DNA polymerase synthesis in hairpin extension assays, among all Taq pol variants analyzed, these four enzymes were at the bottom of the scale. PCR efficiency was quite high in all cases, 1.82 to 1.99, except for S515D (1.73) and F667A (1.59). As for mutants S515F and M747D, they were fully PCR-active.

**TABLE 1:** Results of multiparametric experimental testing of 18 Taq pol mutants and of the WT enzyme.

Enzyme	RT, 90 nt cDNA	RT, 116 nt cDNA	RT, 201 nt cDNA	RT, 526 nt cDNA	k/k_wt_	Fidelity, nt/error	dT/dU rate	T opt approx, °С	LNA1 delay, fold	LNA2 delay, fold	LNA3 delay, fold	K_d_, nM	k_Cat_(dT), s^-1^	k_Cat_(dU), s^-1^	Antibody blocking	Aptamer blocking
E507K	−0.9	−1.89	4.65	6.85	0.25	5,205.19	1.00	64.00	2.78	1.19	4.41	3.38	27.66	23.60	Absent	Strong
M747Q	−0.81	−0.33	1.12	8.89	0.50	4,985.04	1.29	63.00	5.15	1.30	9.11	4.41	24.75	24.89	Absent	Strong
D578K	−0.73	1.22	0.55	6.76	0.15	5,140.39	1.16	62.75	2.16	1.44	6.81	4.20	19.39	10.57	Strong	Strong
D578N	−0.53	0.93	0.76	9.9	0.38	4,677.34	1.08	63.5	3.92	1.41	7.61	4.26	23.86	24.85	Absent	Strong
E507Q	−0.34	0.72	2.76	9.98	0.40	5,633.62	1.13	64.00	4.76	1.51	8.38	4.78	33.94	27.92	Absent	Strong
D578W	1.09	1.95	1.39	7.56	0.17	5,440.88	0.98	63.50	2.40	1.30	5.03	4.99	19.04	19.17	Strong	Strong
S515N	2.37	2.63	11.7	N/A	0.58	7,742.98	1.29	69.75	8.52	1.31	11.19	6.71	26.83	29.12	Strong	Strong
E507D	2.53	4.61	7.63	N/A	0.51	6,644.88	1.32	66.50	4.50	3.09	13.31	21.44	36.71	30.31	Absent	Strong
WT Taq	4.44	5.34	15.29	14.33	1	5,986.21	1.29	72.25	4.68	1.66	10.67	6.19	35.56	32.99	Strong	Strong
F667Y	5.34	7.59	13.48	N/A	0.30	8,433.00	1.34	67.75	9.44	2.28	22.43	11.66	15.35	11.84	Weak	Strong
S515D	6.09	9.53	9.46	N/A	0.14	14,600.8	1.18	74.00	7.36	2.618	15.33	80.71	9.22	9.43	Strong	Strong
M747E	6.19	8.42	11.39	N/A	0.32	7,543.12	1.21	71.00	8.64	4.98	19.42	25.53	17.37	16.30	Strong	Weak
F667M	9.75	11.06	14.65	14.16	0.13	13,759.40	2.11	70.00	12.20	2.76	>30	8.79	7.00	1.75	Strong	Strong
H639Y	9.76	10.44	14.88	N/A	0.15	2,707.11	1.86	69.75	11.81	3.05	22.22	7.46	9.56	3.16	Strong	Strong
H639A	10.51	11.78	13.96	N/A	0.06	14,470.5	2.61	68.00	3.70	2.09	11.25	8.14	1.94	0.23	Strong	Strong
H639F	11.88	12.81	17.87	N/A	0.04	8,614.00	2.09	61.75	7.36	3.06	13.68	6.55	1.75	0.10	Strong	Strong
M747D	13.2	16.06	16.23	N/A	0.06	15,244.40	2.00	69.00	14.04	4.08	>40	29.63	4.61	2.18	Strong	Absent
S515F	14.05	15.48	N/A	N/A	0.07	6,114.36	1.66	72.75	1.34	2.23	9.31	211.11	10.00	10.00	N/A	Weak
F667A	16.12	18.05	N/A	N/A	0.02	42,014.30	2.38	71.00	10.36	2.14	23.43	11.14	0.39	0.12	Strong	Weak

Enzymes that manifested sufficient RTase activity are labeled in red. RTase activity was assessed as the difference in Cq values in the synthesis of each cDNA between a Taq pol mutant and the p66 RTase. The delay caused by the presence of LNA in a model substrate was estimated as the difference in reaction rates between a substrate containing an LNA nucleotide and the control substrate (without such a modification); nt, nucleotides; N/A–below detection limit.

Eight enzymes showed higher RTase activity compared to the WT, and for six of the eight (two mutants in the thumb domain, three mutants in the palm domain, and one mutant in the finger domain), this improvement was substantial. The considerable increase in RTase activity in these proteins was detected both by RT-PCR and by elongation of oligo (dT) primers on a poly (rA) substrate.

The enzymes with noticeably enhanced RTase activity showed (see Table 1) the following.

• higher catalytic constants for both dT-containing and dU-containing dNTP mixes;
• fidelity of DNA-dependent DNA polymerase activity comparable to that of the WT or lower; a decreased (except for M747Q) dT/dU rate;
• stronger affinity (lower K_d_) for a DNA substrate as compared to the WT enzyme;
• reduced negative effects of an LNA in the model substrates, regardless of its position;
• a lowered temperature optimum of DNA-dependent DNA polymerase activity.

In all cases, the cDNA synthesis ability of mutant enzymes decreased markedly with increasing target length. ΔCq values of all mutant enzymes versus the p66 showed the highest correlation between the 90-nt and 116-nt targets (*R* ^2^ = 0.99). With the shortest fragment, some Taq mutants outperformed the p66. Two enzymes failed to produce detectable 201-nt cDNA, and regarding 526-nt cDNA, it could be generated only for eight enzymes out of the 19, always with a substantial lag behind the p66. The ability to process the longest target clearly correlated with catalytic constants and negatively correlated with the LNA-mediated delay and K_d_.

Some mutations led to the loss of the blocking of the enzyme by the antibody or the DNA aptamer. Namely, the M747Q mutation resulted in a loss of binding to the antibody while affinity for the aptamer was retained. In contrast, M747D led to loss of binding to the DNA aptamer but had no effect on the blocking by the antibody.

Utilizing the initial version of our model, which was trained on the dataset comprising the 19 data points, we made predictions of various properties of Taq pol mutants while specifically targeting those within a conservative trust radius of three amino acid substitutions away from the WT sequence. This constrained approach was chosen because our primary objective was to develop industrial candidate enzymes, which must meet multiple stringent requirements to function effectively. Therefore, we focused on mutations at specific sites identified in (Raghunathan and Marx, 2019). As a result, over 18 million Taq pol mutants were assessed under our prediction model.

### Selection and testing of the second and third batches of mutant polymerases

In further experiments, for resource-saving reasons, we reduced the set of experimentally estimated parameters: 1) excluded the LNA-2 assay, which simulated the incorporation of an LNA nucleotide into a newly synthesized strand (this was outside the scope of our purposes); 2) excluded the LNA-1 assay as redundant in relation to the LNA-3 assay (the same characteristic and a less pronounced effect); excluded k_Cat_(dT) and k_Cat_(dU) measurement because of low throughput (the same features of Taq pol mutants were indirectly evaluated by higher-throughput assays); 4) skipped the assessment of the temperature optimum, which in our experiments showed too high variation. Of the remaining parameters, RTase efficiency on 90-nt and 116-nt templates, the effective reaction rate constant, fidelity, PCR efficiency, the dT/dU rate, k_Cat_(dT), and k_Cat_(dU) (based on the experimental results obtained on the first batch of enzyme variants), and probability of enzyme blocking by the antibody or by the DNA aptamer were chosen as independent parameters for model training. The LNA-3 substrate delay, RTase efficiency on the 201-nt and 526-nt templates, and RTase activity measurements with the oligo (dT)-poly (rA) complex were not used for the training (because these characteristics could not be reliably evaluated by our methods in all enzymes) but were monitored in the wet-lab experiments.

When selecting candidates for enhancing RTase activity, we were guided by the following criteria: k_Cat_(dT) and k_Cat_(dU) at least 20 nM, PCR efficiency not less than 1.85, the dT/dU rate not more than 1.5, the error rate not greater than 1/3,000, the effective reaction rate constant ≥0.2 of the WT, and the probability of no blocking with the antibody or DNA aptamer not more than 0.1. The remaining characteristics were disregarded in the selection.

To assess the predictive power of the newly developed algorithm and for its possible further refinement, we selected and synthesized another 13 mutant Taq pols on the basis of both the model predictions and literature data. The logic of their choice was as follows.

• to include new sites for a single substitution as compared to those used in the initial model training; the new substitutions had to have a positive impact on the quality of subsequent predictions (R660S and D732N);
• to include two- or three-substitution mutants expected to evolve in opposite directions according to the model predictions: enhanced RTase activity (E507K-D732N, D578H-M747Q, E507Q-S515N-D578S, E507Q-D578S-I614M, E507Q-D578S-R728Q, D578S-R728Q-M747Q, and D578S-R728Q-M747V) and higher fidelity (V586N-M747T, S515N-M747S, S515D-K540M, and F667I-R728N-M747S);
• to include some mutants for which there were literature data on the properties of interest to us [R660S: enhanced allele-specificity and Sanger sequencing quality (Yoshida et al., 2001; Li, Mitaxov, and Waksman, 1999) and D732N: stronger RTase (also predicted by our model) and strand displacement activities (Barnes, Zhang, and Kermekchiev, 2021)].

According to the obtained experimental findings, the predictive model was refined and a third batch of mutant enzymes was prepared for its adjustment; these enzymes included additional new substitution positions and combinations thereof. This batch contained 15 enzymes. For eight of them (E507K-D578E, R573K-D578N, D578N-V783I, D578Q-D732N, E708Q-D732N, E507R-R573K-E708Q, E507K-A570G-M747Q, and E507K-A570T-M747Q) RTase activity enhancement was predicted by the model. For the other seven, literature data about various useful properties were available, e.g., increased allele specificity [R660V (Drum et al., 2014) and E507K-R536K-R660V (Lim et al., 2022)], cold sensitivity [I707L and E708D (Kermekchiev, Tzekov, and Barnes, 2003)], resistance to PCR inhibitors [E708L (Kermekchiev et al., 2009) and E742K-A743R (Yamagami et al., 2014)], and resistance to PCR inhibitors along with concurrent cold sensitivity [E626K (Kermekchiev, Tzekov, and Barnes, 2003)].

Results of the testing of the second and third batches of enzymes are given in Table 2. The information on changes in physical and chemical properties (volume, charge, aromaticity, and polarity), resulting from different substitutions tested, is provided in Supplementary Table S3.

**TABLE 2:** Results of multiparametric experimental testing of 28 Taq pol mutants.

Enzyme	RT, 90 nt cDNA	RT, 116 nt cDNA	RT, 201 nt cDNA	RT, 526 nt cDNA	k/k_wt_	Fidelity, nt/error	dT/dU rate	LNA3 delay, fold	K_d_, nM	Antibody blocking	Aptamer blocking	Round	Literature data
E507Q-D578S-I614M	−1.01	−1.08	−0.87	8.9	0.50	1,921.59	1.39	1.87	1.17	Absent	Strong	II	
E742K-A743R*	−0.95	−0.75	0.66	3.42	0.24	4,051.98	1.13	1.43	0.51	Weak	Weak	III	Inhibitor resistance
E507K-D732N	−0.53	−0.67	0.08	12.22	0.17	3,696.37	0.97	1.91	1.69	Absent	Strong	II	
D732N*	−0.51	−0.03	5.35	N/A	0.95	4,328.99	1.15	4.12	2.60	Strong	Strong	II	Strand displacement, RT
D578H-M747Q	−0.47	0.3	0.2	N/A	0.25	3,634.96	0.88	3.08	3.29	Strong	Strong	II	
E507K-D578E	−0.41	0.32	2.14	12.54	0.54	4,636.97	0.93	3.80	1.07	Absent	Strong	III	
D578N-V783I	−0.15	−1.54	−0.37	10.57	0.36	2,537.75	1.01	2.23	2.19	Strong	Strong	III	
E507K-A570T-M747Q	−0.09	−1.4	0.42	8.29	0.51	4,147.97	1.09	2.85	1.53	Absent	Strong	III	
E507K-A570G-M747Q	−0.01	−1.56	0.34	7.82	0.79	4,459.81	0.89	3.20	5.15	Absent	Strong	III	
E507Q-S515N-D578S	0.89	1.59	3.06	12.16	0.40	3,966.74	1.95	2.33	2.91	Absent	Strong	II	
D578Q-D732N	1.23	1.46	2.92	18.32	0.21	4,334.18	1.07	5.42	0.80	Strong	Strong	III	
E708Q-D732N	1.34	0.14	3.58	7.17	0.77	4,382.99	1.06	5.77	4.33	Strong	Strong	III	
E507K-R536K-R660V*	2.14	4.36	8.01	N/A	0.91	13,051.67	1.09	>40	0.79	Absent	Strong	III	Allele specificity
S515N-M747S	2.84	1.49	3.01	8.9	0.43	4,150.20	0.68	4.47	1.93	Strong	Strong	II	
V586N-M747T	3.05	3.81	6.69	N/A	0.48	5,192.41	0.84	3.85	14.09	Strong	Strong	II	
E708L*	3.32	4.44	6.97	N/A	0.86	5,164.52	1.23	8.27	6.82	Strong	Strong	III	Inhibitor resistance
R660S*	4.07	6.38	9.56	N/A	0.70	10,996.46	1.01	>30	10.64	Strong	Strong	II	Allele specificity, decreased transition rate
E708D*	5.14	5.99	8.75	N/A	1.40	5,000.88	1.11	8.78	4.51	Strong	Strong	III	Cold sensitivity
E626K*	5.26	5.47	8.87	24.13	0.25	5,228.77	0.69	12.14	5.74	Strong	Strong	III	Inhibitor resistance, cold sensitivity
I707L*	6.21	5.95	11.14	N/A	0.71	7,441.63	1.61	>40	8.54	Strong	Strong	III	Cold sensitivity
R660V*	6.96	10.07	12.58	14.01	1.04	15,402.44	1.69	>40	6.21	Strong	Strong	III	Allele specificity
E507Q-D578S-R728Q	7.21	10.66	11.83	N/A	0.31	6,889.79	2.28	>40	6.29	Absent	Strong	II	
D578S-R728Q-M747Q	8.04	8.9	9.63	N/A	0.48	5,946.68	2.10	>40	10.35	Strong	Strong	II	
D578S-R728Q-M747V	8.20	11.54	14.2	13.78	0.24	6,346.44	2.49	>40	13.81	Strong	Strong	II	
S515D-K540M	9.90	11.49	13.93	N/A	0.01	28,816.50	2.03	N/A	>200	Strong	Strong	II	
R573K-D578N	11.33	14.53	N/A	N/A	0.08	7,828.28	3.57	>40	4.33	Weak	Weak	III	
E507R-R573K-E708Q	12.18	16.14	N/A	N/A	0.04	13,458.91	2.65	>40	0.96	Absent	Strong	III	
F667I-R728N-M747S	14.75	N/A	N/A	N/A	0.02	22,506.31	2.64	>40	14.38	Strong	Strong	II	

Enzymes that exerted sufficient RTase action are labeled in red. Enzymes selected based on literature data are marked with asterisks. RTase activity was assessed as the difference in Cq values in the synthesis of each cDNA between a Taq pol mutant and the p66 RTase. The delay caused by the presence of an LNA nucleotide in a model substrate was estimated as the difference in reaction rates between the substrate containing LNA and the control substrate (without such a modification). Round corresponds to experimental evaluation round (see Figure 1). N/A–below detection limit.

**FIGURE 3:**
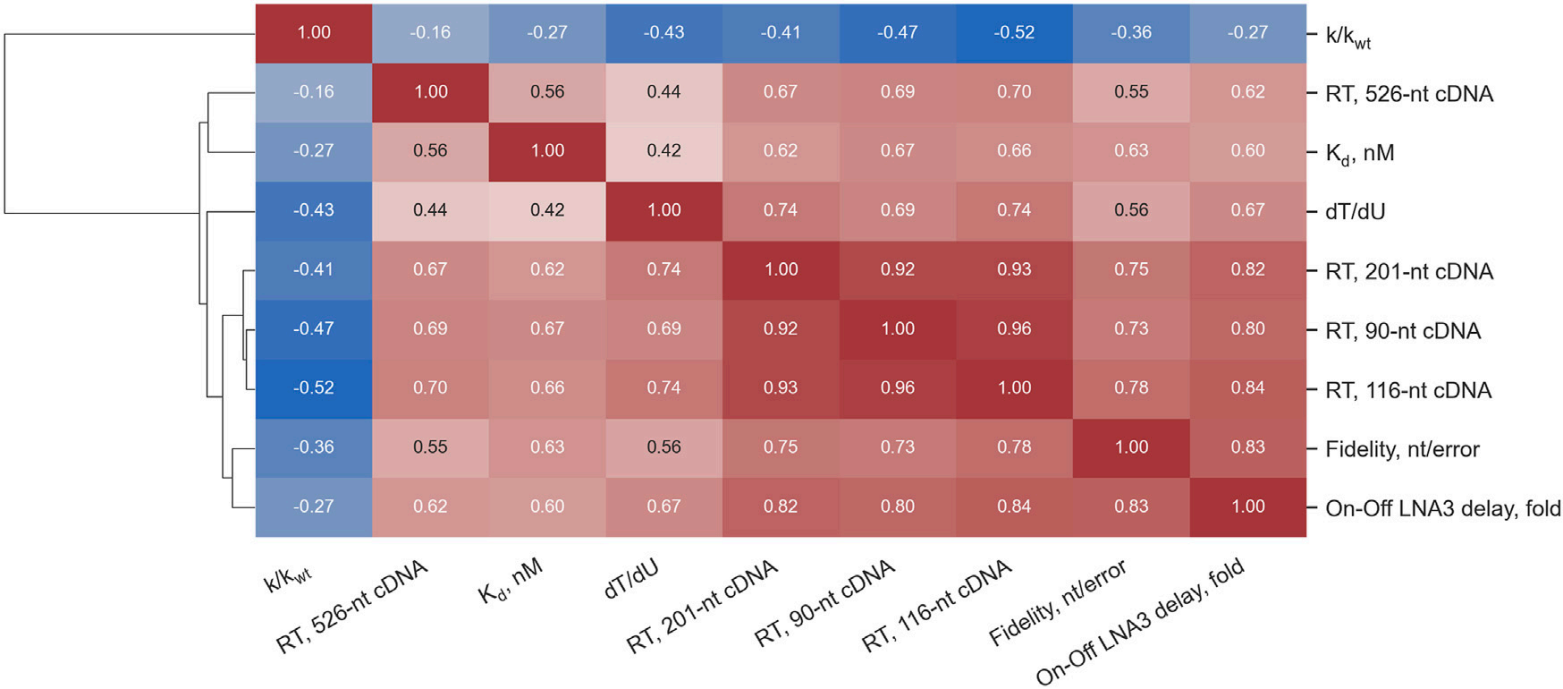
Spearman’s correlations between the parameters measured for 46 Taq pol mutants and the WT enzyme. The color scale varies from deep blue for highly negative correlation coefficients to red for highly positive ones.

In Figure 3, Spearman’s correlations between the parameters experimentally measured for all 46 Taq pol mutants and the WT are presented.

Overall, the correlation between predicted and experimentally confirmed RTase activities for all enzymes was quite high (see Supplementary Figure S2). Unexpectedly, both mutants containing substitutions in codon 728 did not exert sufficient RTase action. Vice versa, mutant E742K-A743R, predicted to have low RTase activity, manifested its enhancement.

Again, the predictive model was refined based on the addition of the new experimental findings. This enhanced model enabled *in silico* screening of over 18 million mutated Taq pol variants. The results of our final biological activity predictions for mutated Taq polymerases can be accessed at https://huggingface.co/datasets/nerusskikh/taqpol_insilico_dms.

Altogether, our search identified 18 Taq pol mutants with substantially enhanced RTase activity as compared to the WT. In all cases, the cDNA synthesis ability of mutant enzymes declined markedly with increasing target length, and this feature disadvantageously distinguished them from native RTases. In this regard, when analyzing the second and third batches of enzymes, we continued to separately monitor the performance of mutants on four RNA templates of different lengths as four independent characteristics and to utilize two of them for training the model. Some enzymes showed improved RTase activity on longer templates as compared to the best enzymes from the first batch.

### Fidelity of DNA-dependent DNA-polymerase activity

**FIGURE 4:**
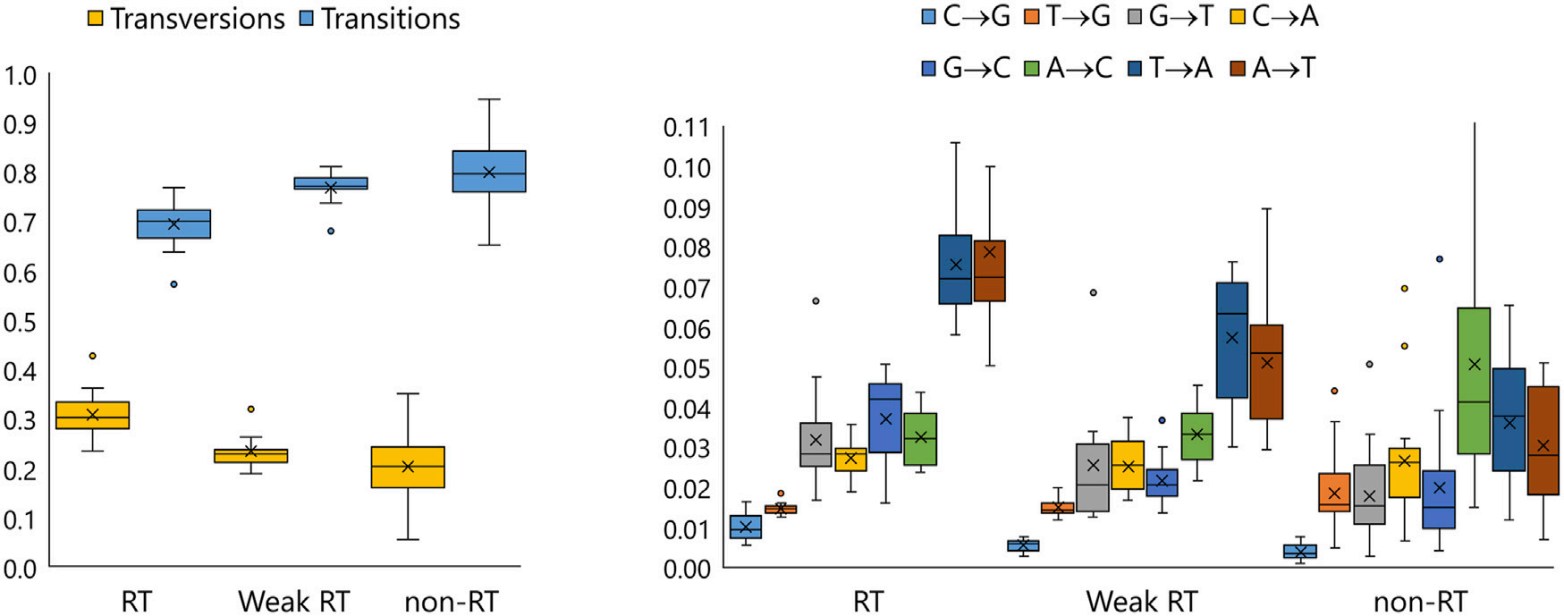
Box-whisker plots representing relative summarized frequencies of transitions and transversions (left) and relative frequencies of specific transversions (right) in DNA-dependent DNA polymerase-driven synthesis by enzymes with sufficiently enhanced RTase activity (RT, n = 18), enzymes with extremely low RTase activity (non-RT, n = 19), and enzymes intermediate in terms of this characteristic, including the WT (Weak RT, n = 11).

Values of overall fidelity of mutant enzymes and of the WT are listed in Tables 1, 2, and a more detailed view (absolute and relative frequencies of different transitions and transversions) is given in Figure 4; Supplementary Table S4; Supplementary Figure S3.

This parameter varied widely in the total collection of enzymes. For all enzymes with enhanced RTase activity, it a) was slightly reduced compared to the WT but is comparable to the values known for HIV-1 RTase (Kati et al., 1993), and b) featured an increase in ratios of transversions to transitions, especially substitutions A- > T and T- > A. The increase in accuracy followed the opposite trend: it matched an increase in the proportion of transitions (*R* ^2^ = 0.73). Not only the overall frequency of transitions and transversions (see Supplementary Table S4) but also the proportion of various substitution types varied among the enzymes, in some cases following rather different patterns (see Supplementary Figure S3).

### Taq pol mutants with enhanced RTase activity in single-tube TaqMan RT-PCR

**FIGURE 5:**
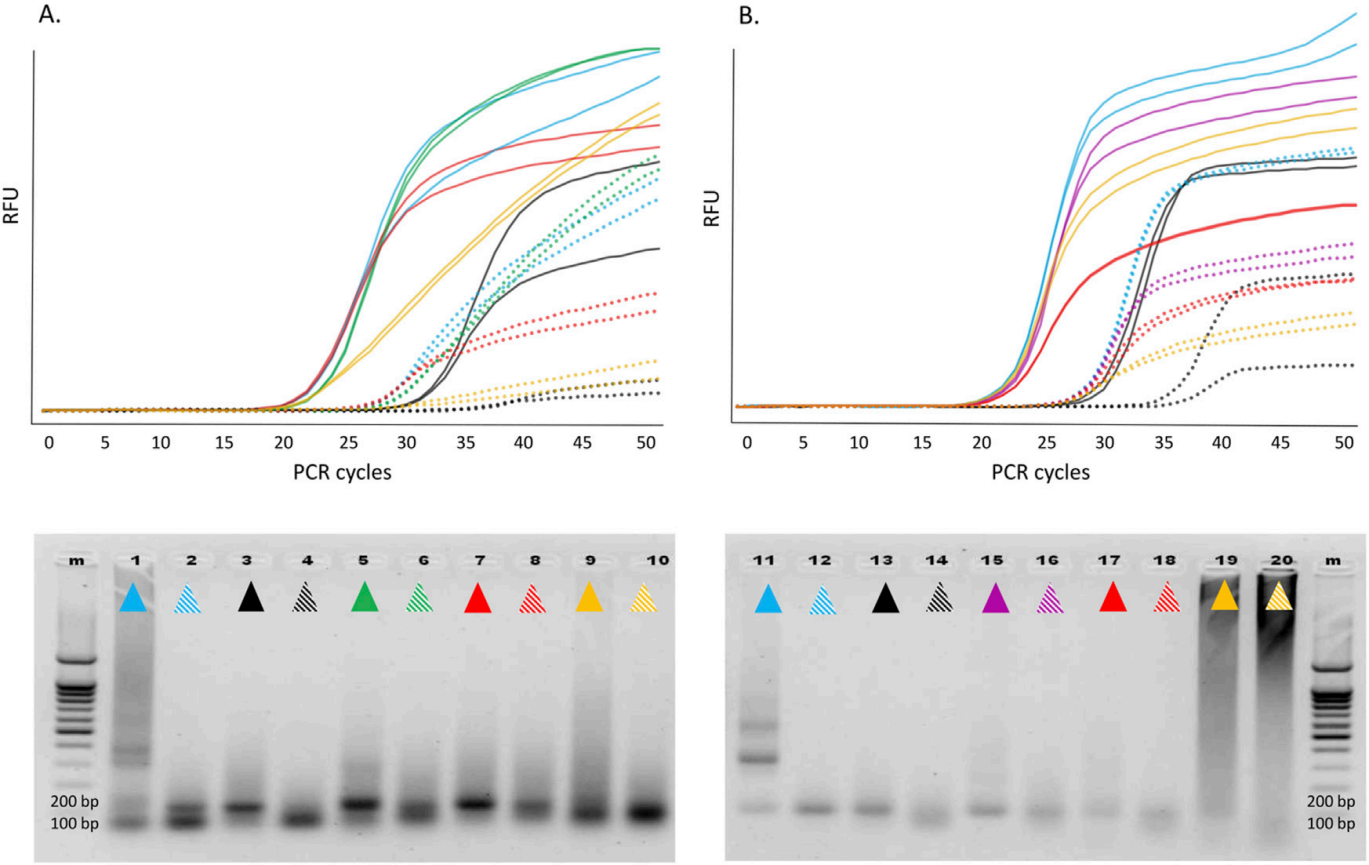
Above: Real-time RT-PCR involving various Taq pol mutants in single-enzyme reactions with a synthetic transcript containing the 116-nt hMPV sequence **(A)** and the 90-nt HPIV sequence **(B)**. Color indication: WT Taq pol, WT/p66 mix, E507Q, D578N, E507Q-D578S-I614M, E507K-A570G-M747Q. For each transcript, two dilutions were analyzed, of which the second (dashed lines) is 1/100 of the first (solid lines). Below: 2% agarose gel electrophoresis of the reaction products (one well per dilution, the color indication as above). Left, hMPV; right, HPIV. M, marker. RFU, relative fluorescence units.

The 12 selected candidate enzymes were tested as described in the *Methods* section (Figure 5).

The findings were interpreted with respect to two parameters: Cq reflects integral efficiency of RT and cDNA amplification, and the amplitude and shape of the fluorescence curve mirror the 5′ exonuclease activity resulting in the cleavage of the labeled probe. All the 12 candidates expectedly outperformed the WT as RTases. As compared to the p66/WT mixture, all these candidates yielded comparable or even lower Cq values, indicating that sensitivity was comparable between the single enzyme mode and two-enzyme mode of target RNA detection. The shapes and amplitudes of the fluorescence curves as well as PCR efficiency of the generated cDNA in the single-enzyme reactions were comparable to those of the control two-enzyme reactions. Nonetheless, in some single-enzyme reactions, we observed a high level of low-molecular-weight and/or high-molecular-weight off-products, which negatively affected the shape and slope of the kinetic curve, especially during HPIV2 RNA detection. In our experience, this phenomenon is characteristic of enzymes with strong K_d_ and can be improved by optimizing the reaction conditions. Indeed, an increase in KCl concentration and pH of the reaction mixture led to a reduction in the amount of off-products, an improvement in the shape of the kinetic curves, and clear-cut electrophoretic bands when enzymes E507K-D732N and E507Q-D578S-I614M were used (data not shown).

## Discussion

It has long been known that WT Taq pol has *in vitro* RTase activity (Jones, and Foulkes, 1989; Tse and Forget, 1990). In numerous papers, it has been demonstrated that this activity can be significantly enhanced by modification of reaction conditions or by introduction of amino acid substitutions at various positions (see refs. above). In our study, we deliberately limited the number of analyzed amino acid positions, thereby radically narrowing the space of possible mutants. Nevertheless, our search identified sufficient number of Taq pol mutants having considerably boosted RTase activity combined with a set of other desirable characteristics. Thus, our approach aimed at generating a comprehensive dataset to train predictive models in balancing resource constraints showed sufficient performance to find promising candidates.

Our attempts to leverage recent advancements in protein modeling involving PLMs were thwarted by hardware-related constraints. Although we managed to fine-tune only the last six layers of ProtT5-XL, we anticipate that the method’s efficiency may be improved through full-model fine-tuning or the use of larger PLMs. Parameter-efficient fine-tuning aroused considerable interest in recent years owing to its potential to facilitate fine-tuning of larger models, such as ProtT5-XXL, on the same hardware (Han et al., 2024). Authors of ref. (Schmirler et al., 2024) provide evidence that low-rank adaptation (LoRA) procedures enhance the ability of PLMs to navigate mutational fitness landscapes, indicating that parameter-efficient fine-tuning may further improve model performance without excessive computational resources.

The use of more advanced or specialized architectures may also enhance our predictive capabilities. Multimodal models like ProstT5, which take both sequence and 3D structure data as inputs, can provide more informed predictions. Although 3D structural information is implicitly present in sequence-derived embeddings, explicit incorporation of structural data can provide additional insights. Moreover, ProteinNPT, as described in ref. (Notin et al., 2023), learns joint representations of full input batches of protein sequences and associated property labels, thereby making possible a prediction of single or multiple protein properties, novel sequence generation via conditional sampling, and iterative protein redesign cycles through Bayesian optimization.

When proteins are designed, it is crucial to consider multiple properties, especially in industrial settings. As a mutation radius increases, it quickly becomes impossible to exhaustively explore the search space thus necessitating a search for algorithms that prioritize candidate mutations for inference. Discrete optimization methods such as genetic algorithms or Markov Chain Monte Carlo, as described in (Biswas et al., 2021), are required for navigating the vast search space and exploring enhanced enzymes that are a couple more mutations away from the WT. This multicriterion optimization is computationally challenging, particularly because some properties entail trade-offs. Finding an optimal balance under such circumstances is an unsolved problem that prevents achieving desired characteristics in industrial applications. Furthermore, restricting the number of amino acid substitutions analyzed per protein to three is of course a serious limitation here, which definitely lowers the probability of finding an optimal enzyme. A number of examples are known where the best properties of a bioengineered Taq pol modified to enhance RTase function have been achieved via introduction of more amino acid substitutions, e.g., TaqM1 (L322M-L349M-S515R-I638F-S739G-E773G) (Marx et al., 2010) or RT-KTq (L459M-S515R-I638F-V669L-M747K) (Aschenbrenner and Marx, 2016).

Another important topic is zero-shot methods for selecting initial datasets when experimental data are unavailable. These procedures typically are based on the likelihood of a sequence according to a PLM. Authors of ref. (Meier et al., 2021) provide details on how such approaches can be implemented effectively to identify promising candidates for initial testing, thereby reducing the experimental burden and accelerating discovery. Although this strategy is outside the scope of our study (because we relied on literature data), it is crucial for similar projects in general.

One of the significant factors that ultimately influenced the accuracy of the predictive model, we considered the suitability of wet-lab experimental data for training this model both in terms of their reproducibility and with regard to the width of the range of values obtained. We focused on methods for assessing the characteristics that could be experimentally evaluated over a wide range of values, ranging from negligible to high, as opposed to categorical variables (all-or-none). Thus, for all RTs and PCRs, we chose conditions that, in our experience and according to the literature, contribute to increased enzymatic activity at the cost of an increased frequency of nucleotide misincorporations: high concentration of trehalose, dNTPs, and magnesium ions. Under these conditions, we, for example, achieved full PCR activity from mutants S515F, H639A, F667A, and M747D, in contrast to their performance in the study (Raghunathan and Marx, 2019). Also, in our experiments, the wild-type Taq was able to synthesize cDNA longer than 500 nucleotides (albeit with low efficiency), whereas, for example, in refs. (Marx et al., 2010; Raghunathan and Marx, 2019), it failed to extend a primer hybridized to a complementary RNA strand for more than 2-7 nucleotides. Accordingly, the methodology that we applied to the estimation of RT activity via a comparison of RT efficiency among specific RNA templates of different lengths and structures by real-time RT-PCR turned out to be much more informative than traditional direct recording of reaction kinetics through elongation of oligo (dT) primers on a poly (rA) substrate. The latter option helped us to distinguish the WT from the enzymes with sufficiently enhanced RT activity but not to stratify them among themselves (data not shown): the low melting temperature of oligo (dT)/poly (rA) duplex prevented obtaining the good-quality kinetic curves at the temperatures optimal for RTase activity of the mutants (>60°C).

These observations reinforce the robustness of our experimental setup in detecting even marginal activities, which is valuable for understanding the gradient of functional impact across mutations. We should emphasize that the RT and PCR conditions we used had default settings, i.e., we did not adapt them to the tested enzymes; this approach could further improve the quality of the obtained data. At the same time, a negative consequence of choosing sparing conditions could be the loss of valuable information associated with the loss of gradation of the property displayed by different mutants if this property is significantly manifested in the prototype enzyme. Thus, in our assays, all the tested enzymes were able to cleave 5′-fluorescently labeled probes, albeit with different signal amplitudes. Although this was a desirable outcome and a criterion for candidates to be selected, the absence of mutations dramatically affecting 5′-nuclease activity prevented us from using this parameter to train the predictive model. Therefore, we cannot rule out that some of the candidates selected with it would be devoid of this activity.

**FIGURE 6:**
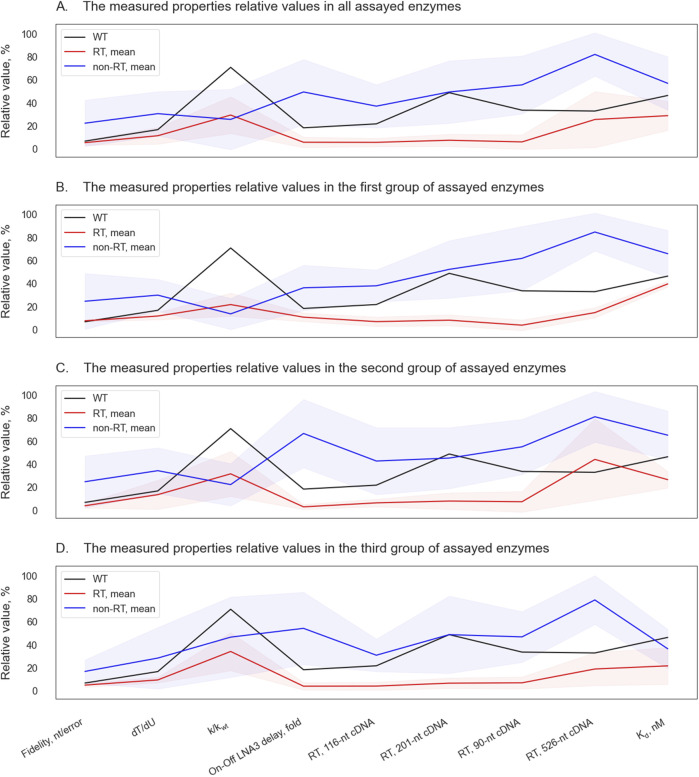
Parallel coordinate plots of the assayed Taq pol mutants and of the WT enzyme. Mean relative values of properties of non-RT enzymes (lacking appreciable RTase activity) are highlighted in blue, and the mean relative values of properties of RT enzymes (having substantial RTase activity) are shown in red. The shaded area denotes standard deviation ranges. Relative values of the WT enzyme’s properties are presented as the black lines. The relative values of measured properties are shown for all the assayed enzymes **(A)**, and separately for enzymes from the first round of experiments **(B)**, from the second **(C)** and from the third round **(D)**.

In our experiments, RTase activity enhancement in Taq polymerase strongly correlated with broadened substrate specificity. Correlations between different parameters, identified in the first enzyme batch, persisted in subsequent ones (Figure 6): the enhancement of RTase activity was accompanied by a general trend toward a diminished dT/dU rate, lower fidelity, lower K_d_ values, and reduced negative effects of LNA modifications (with the exception of D732N), whereas the enhancement of fidelity followed the opposite trend.

The fact that this correlation was expressed for the entire prediction space (data not shown) and did not change for the different stages of experimental attestation indicates that we are not dealing with a self-fulfilling prediction, but with a real pattern that may have a mechanistic explanation. This pattern may be beneficial for the development of tools for biotechnology and molecular genetic studies. Thus, the increased ability to use LNA substrates enhances the design of oligonucleotide primers and fluorescently labeled probes in terms of providing the necessary specificity and adjusting annealing temperatures. In this regard, it was desirable for us to obtain mutants that allow LNA substrates to be processed at least no worse than the wild-type enzyme. To our pleasure, the increase in RTase activity in most cases not only did not lead to a deterioration of this parameter, but also provided additional tolerance to LNA substrates. Another useful property also found to be associated with an increase in RTase activity–an improved efficiency of adjusting deoxyuracyl–is useful for molecular diagnostic tools since it allows more effective use of uracyl-DNA glycosylases to prevent carry-over contamination, as well as fine-tune multiplex reactions.

However, the interdependence of these properties raises the question of whether, in principle, the combination of some required characteristics for one enzyme is achievable if the enhancement of one of them entails weakening of the other, and vice versa. In our study, we faced two such trade-offs: reduced fidelity and catalytic efficiency for longer targets in all enzymes with enhanced RTase activity, and, vice versa, the negligible RT function in other mutants with increased fidelity. In our study, we did not set the goal of obtaining the Taq pol variant with the combination of enhanced RTase activity and high fidelity. We do not rule out that such a task can be solved by involving other sites for directed mutagenesis. In our study, all candidates with enhanced RTase activity had slightly reduced fidelity, but not reduced so drastically as to prevent their use in routine RT-PCR applications. In our experience and according to the literature, such a fidelity decrease can be compensated by parameters of the reaction mixture composition, such as the concentration of magnesium ions, dNTPs, monovalent cations, or the use of additives (Eckert and Kunkel, 1990; Xue et al., 2021).

As for the target length–dependent decline of cDNA synthesis efficiency in all mutants, it may be explained by their low processivity on the RNA templates, lack of strand displacement (SD) activity, non-optimal reaction temperature, a high degree of product inhibition, or other factors or combinations thereof. Wild-type Taq pol is known for the extremely low SD activity compared to the mesophilic reverse transcriptases but this activity can be enhanced through the introduction of mutations (Ignatov et al., 2014; Barnes, Zhang, and Kermekchiev, 2021). In our sample of tested enzymes, only D732N displayed pronounced SD activity (which was confirmed by our experiments; data not shown). However, in our hands, in the synthesis of longer cDNAs, D732N was outperformed by E507Q-D578S-I614M polymerase that did not display SD activity. Further elucidation of the key mechanisms underlying the length-dependent reduction in cDNA synthesis by Taq pol mutants is needed to help identify a way to overcome this limitation.

Judging by our results, the dissociation constant of a polymerase and a primer-template complex could be a key parameter responsible for the substrate specificity expansion: it was mutants with lower K_d_ that showed such expansion. Moreover, although the K_d_ value was not our criterion for the selection of candidates for wet-lab experiments, the selection for K_d_ actually occurred coupled with the selection for RTase activity (see Figure 3). A simple explanation is that the stronger binding of mutant enzymes to various substrates enables reactions with these substrates, in contrast to the WT, for which such reactions are inhibited/prevented by the weak affinity for the substrates. On the other hand, our analysis of the same set of experimental data suggests that “1D” selection aimed at increasing K_d_ of a polymerase and the primed template may not be effective enough to find the best candidates for different biotechnological tasks. This is due to the fact that effects of different “K_d_-lowering” mutations on features of interaction with one or another type of substrate were different even among enzymes with high DNA-dependent DNA polymerase activity. For example, E708D has lower K_d_ compared to the WT but showed no improvement of RTase activity, whereas V586N-M747T has higher K_d_ but manifested moderately increased RTase activity and better tolerance to an LNA substrate. This is not surprising because the effects of these mutations on enzyme–substrate interactions can have dissimilar mechanisms. For instance, the thumb domain, which includes E507, is involved in interactions with the upstream duplex of an overlapping substrate (Ma et al., 2000). E507 is located in the primer–template-binding site where mutations are expected to modulate DNA-binding affinity. E507K stabilizes the Taq pol–DNA binary complex by forming additional contacts with the distal portion of the primed template (Arezi et al., 2014). S515 is important for α-helix stabilization and makes the nucleic-acid–binding motif more robust (Blatter et al., 2013). D578 (palm) comes into contact with a template strand (Raghunathan and Marx, 2019). E742K and M747K (finger) could be structurally responsible for the formation of a salt bridge with negatively charged template phosphodiester groups located close to aa 739 and 747 or with a nucleotide’s triphosphate group close to aa 817 (Vichier-Guerre et al., 2006). M747K introduces an additional positive charge near the negatively charged RNA template backbone thereby possibly helping to accept an unnatural substrate by enhancing binding. In turn, D732N (finger) seems to be at a distance from primer and template strands in crystal structures (Barnes et al., 2021), and its participation in enzyme–substrate interactions is not fully understood. I614 (finger) contacts an incoming nucleoside-5′-O-triphosphate. Ref. (Patel et al., 2001) indicates that Taq pol tolerates amino acid substitutions at position I614 and that such mutant enzymes retain activity similar to that of the WT, but fidelity is often low. In their experiment, however, nonhydrophilic substitutions, including I614M, did not alter the error rate during DNA synthesis.

The observed influence of tested amino acid substitutions on Taq pol RTase activity can be illustrated with a biplot of a partial least squares (PLS) model (Supplementary Figure S4). The PLS model was trained to predict on the basis of mutation data whether enzymes have RTase activity. When we analyzed some mutations’ frequencies in Taq pol mutants predicted to possess the RTase activity, we noticed that the majority of these proteins contain at least one of such mutations as E507K or E507R, E742Q or E742M or E742H, M747K, I707R or I707K, A570K or A570R (see Supplementary Table S5).

Understanding the “linkage” of effects of individual mutations or combinations thereof on several characteristics of mutant polymerases allows for predicting the properties of enzyme variants, for which these characteristics are unknown or unpublished. For example, the I704L mutation described in the literature as leading to cold sensitivity (Kermekchiev et al., 2003), in our experiments (in full accordance with the predictive model) also led to higher K_d_ values, an RTase activity lower even compared to the WT, diminished efficiency of dUTP incorporation, and low tolerance to a hairpin LNA-containing substrate. All three mutants possessing increased allele specificity according to literature data [R660S, R660V, and E507K-R536K-R660V (Drum et al., 2014; Lim et al., 2022)] expectedly showed elevated fidelity and a greater delay in synthesis on the LNA templates as compared to the WT enzyme. At the same time, a downside of this “linkage” may be unexpected “side effects” of selection for some useful property. In our work, these were pronounced attenuation of the negative influence of an upstream LNA nucleotide and a decrease in a temperature optimum during selection for greater RTase activity. We have no doubt that the effects of amino acid substitutions and their combinations on other useful properties of enzymes, for example, tolerance to PCR inhibitors, DNA lesion bypass, or the ability to incorporate fluorescently labeled monomers, can be connected and predicted *in silico*. This accomplishment should make it possible to rationally design an enzyme with a preselected combination of properties without the need to validate *in vitro* a huge number of candidates.

Important limitations of the study should be mentioned. Firstly, some key characteristics (e.g., the Michaelis constant, processivity, and affinity to dNTPs) were not evaluated at all. Secondly, some identified patterns could be attributed to specific reaction conditions. Thirdly, we evaluated only the fidelity of DNA-dependent DNA polymerase activity because we were able to assess this characteristic in the entire set of mutant enzymes. Regarding the fidelity of RNA-dependent DNA polymerase–driven synthesis, it was determined only in a small number of enzymes (data not shown). Fourthly, we established a purely empirical relation of individual mutations or their combinations with properties of the enzyme without examining the physicochemical mechanisms underlying the effects of these mutations. That is to say, the observed decreased extension rate of all enzymes on LNA-containing hairpins may be subject to different interpretations [see (Di Giusto, and King, 2004; Pande, and Nilsson, 2008; Fakhfakh et al., 2015)]. And, in the fifth, we didn’t test the most of the enzymes from the top suggested by the predictive tool, where perhaps significant exceptions to the patterns we postulated could be found.

## Conclusion

Through a screening of a collection of 47 mutant Taq DNA polymerases — 29 of which were selected via our proposed strategy of multiparametric rational design—we were able to identify 18 enzymes that possess orders of magnitude higher RTase activity on all three substrates considered as compared to the wild-type enzyme; 12 of these Taq pol mutants were selected by our AI-based algorithm. The analyzed mutants contain amino acid substitutions affecting 21 positions in all three structural domains of Taq pol. As predicted by our algorithm and subsequently confirmed experimentally, the RTase activity enhancement tends to be accompanied by lower K_d_ values, moderately decreased fidelity, and greater tolerance to noncanonical substrates such as dUTP and/or LNA modifications. Some mutants were effective in single-enzyme RT-PCRs involving cleavage of fluorescently labeled probes or an antibody- or aptamer-mediated hot start. These improvements made the mutants suitable for advanced molecular diagnostic applications, particularly in high-temperature reverse transcription and single-enzyme real-time RT-PCR setups. Therefore, they can provide the basis for the creation of new diagnostic tools such as pathogen RNA detection or gene expression analysis.

We regard our results as proof-of-concept data, not as a final solution even in relation to the problem in question, and we do not rule out the possibility of optimizing our approach toward an analysis of combinations of more mutations, based on the accumulated body of empirical data and the new insights. The fact that all mutants with enhanced reverse transcriptase activity exhibited the two functional trade-offs (reduced fidelity and catalytic efficiency for longer targets) highlight the challenge of optimizing multiple enzymatic properties simultaneously, as improvements in one function can adversely affect others. When new enzymes are bioengineered to solve specific biotechnological problems, it must be borne in mind that enhancing one function may entail inevitable and multidirectional alterations of other functions, sometimes in an unpredictable manner.

Nonetheless, in our study, deep learning models proved to be valuable for guiding our selection of mutations, thereby highlighting good potential of AI-driven approaches in enzyme engineering especially in settings with a relatively small number of experimental studies.

## Data Availability

The datasets presented in this study can be found in online repositories. The names of the repository/repositories and accession number(s) can be found in the article/Supplementary Material.
