# Supplementary material for: Enhancing the reverse transcriptase function in Taq polymerase via AI-driven multiparametric rational design
Source: Front Bioeng Biotechnol. 2024 Dec 10;12:1495267. doi: 10.3389/fbioe.2024.1495267 (PMC11666352; doi:10.3389/fbioe.2024.1495267)
Supplement: Supplementary file 1 [file DataSheet1.zip › Figure S2.PPTX]

## Slide 1
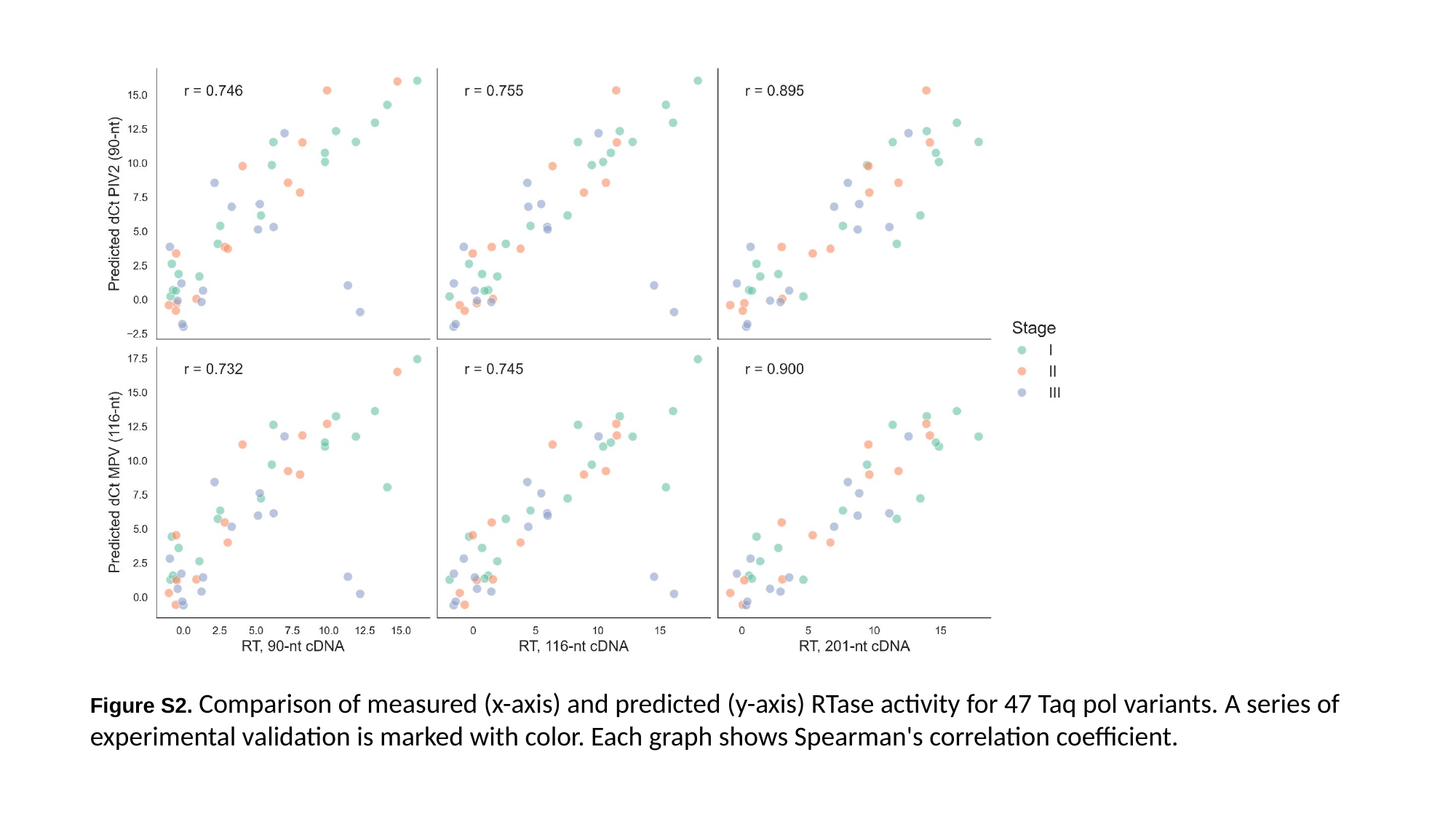

Figure S2. Comparison of measured (x-axis) and predicted (y-axis) RTase activity for 47 Taq pol variants. A series of experimental validation is marked with color. Each graph shows Spearman's correlation coefficient.
