# Supplementary material for: Enhancing the reverse transcriptase function in Taq polymerase via AI-driven multiparametric rational design
Source: Front Bioeng Biotechnol. 2024 Dec 10;12:1495267. doi: 10.3389/fbioe.2024.1495267 (PMC11666352; doi:10.3389/fbioe.2024.1495267)
Supplement: Supplementary file 1 [file DataSheet1.zip › Figure S3.DOCX]

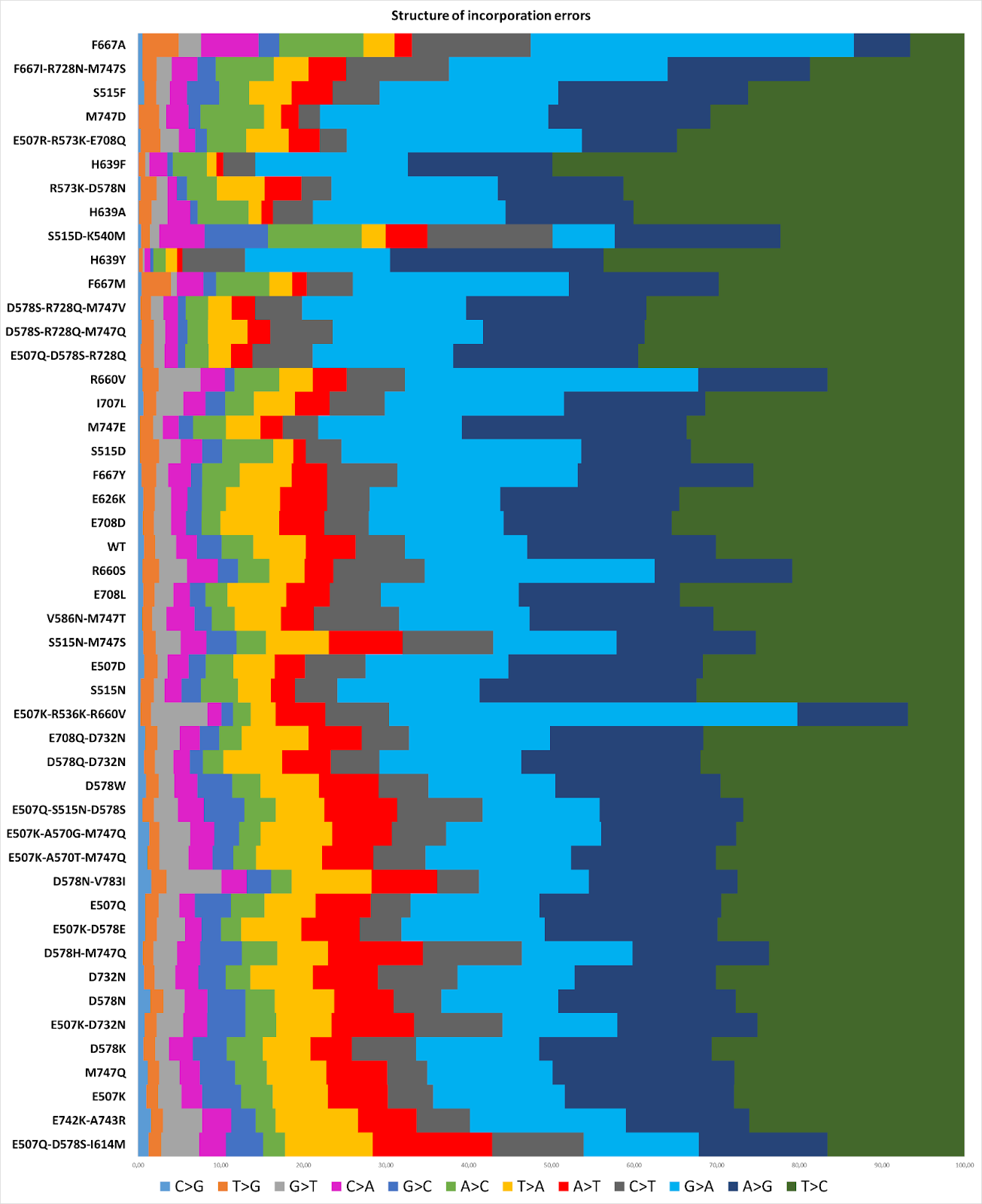


**Figure S3.** Structure of incorporation errors made by different Taq pol variants. The enzymes are stratified from top to bottom by an increasing RTase activity.
