# Supplementary material for: Enhancing the reverse transcriptase function in Taq polymerase via AI-driven multiparametric rational design
Source: Front Bioeng Biotechnol. 2024 Dec 10;12:1495267. doi: 10.3389/fbioe.2024.1495267 (PMC11666352; doi:10.3389/fbioe.2024.1495267)
Supplement: Supplementary file 1 [file DataSheet1.zip › Table S1.DOCX]

**Table S1. Oligonucleotides used in the study.**

**1) Hairpin templates. Bold denotes self-complementary regions. (+T) denotes LNA-T modification. SIMA, Dichloro-diphenyl-fluorescein.**

H-Kd (for Kd determination)

5’-GCTATGCGTGAGCGCT(T-SIMA)TTGCGCTCACGCATAGC

20_60 (for kCatT and kCatU determination)

5’-AAACTCAAGCCTTCCACATATTCTATCCACGACTCTTTACAGGTAGTATAATCTCACTATCGGACTGACTGCGTGAGCGCTTTTGCGCTCACGCAGTCAGTCCG

OnOff1_7G-C_long1 (for kinetic analyses)

5'-ATCTCGCTGTACTCACCGGTTCCGCAGATCGACATACTTATTAACTTATCTATCCTCTTACTTCATCTCATCGATACTTTTGTATCGATGAGATGAAG

LNA 0-3 (for the analysis of LNA-associated effects)

LNA-0 5'-CACTCCACAGGTCGCATTGCTAGCTGGTTAGAGACTAGTAGATCGGAAGAGCCATGAACTCCUACACTCTACGGCTCTTCCGATC

LNA-1 5'-CACTCCACAGGTCGCATTGCTAGCTGGTTAGAGACTAGTAGATCGGAAGAGCCATGAACTCCUACACTCTACGGCTCTTC(+C)GATC

LNA-2 5'-CACTCCACAGGTCGCATTGCTAGCTGGTTAGAGA(+C)TAGTAGATCGGAAGAGCCATGAACTCCUACACTCTACGGCTCTTCCGATC

LNA-3 5'-CACTCCACAGGTCGCATTGCTAGCTGGTTAGAGACTAGTAGATCGGAAGAGCCATGAACTCCUACACTCTACGGCTCTTCCGA(+T)C

**2) Primers and fluorescently labeled probes for RT and PCR. (+T) and (+C) denote LNA-T and LNA-C modifications. ROX, 6-carboxyl-X-Rhodamine. BHQ2, Black Hole Quencher-2. R, purine (A or G). F, forward primer. R, reverse primer. P-probe.**

HBV-F 5'-TGTCTGCGGCGTTTTATCA

HBV-R 5'-AGGACAAACGGGCAACATAC

HBV-P 5'-ROX -CATCCTGCT(BHQ2)GCTATGCCTCATCTTCTTRTTGG-p

HPIV2-F 5'-AAAGAGCAAGAGGCAACCATA

HPIV2-R 5'-CACACCTGGGATTACACCA

HPIV2-P 5'-(ROX)-TCT(+C)CGACCCA(T-BHQ-2)GCAATACTCCATTCTCTC-p

hMpV-F 5'-CTCTACAGGCAGCAAAGCA

hMpV-R 5'-CATTATATTGTTAGATGACCTGGCAA

hMpV-P 5'-(ROX) CC(+C)(+C)ACCT(T-BHQ-2)AG(+C)AGTGTTTGA(+C)C-p

RhV-F 5'-CTAGCCTG(+C)G(+T)GG

RhV-R 5'-GAAACACGGACACCCAAAGTA

RhV-P 5'-(ROX)TC(+C)GGCC(+C)C(T-BHQ-2)GAATGTGGCTAA-p

HIV1-F 5’-GACTCTGGTAACTAGAGATCCCTCAGA

HIV1-R 5'-CTTGGTGTCTCTTATGTCTATCTTTTG

HIV1-P 5’-(ROX)-TCTCTAGCAGTGGCGCCCGAACA-(BHQ2)
